# Remote Access to Urinary Incontinence Treatments for Women Veterans: The PRACTICAL Randomized Clinical Trial

**DOI:** 10.1001/jamanetworkopen.2025.32111

**Published:** 2025-09-16

**Authors:** Alayne D. Markland, Karen M. Goldstein, T. Mark Beasley, Emily Malone Boyd, Lisa Zubkoff, Ursula A. Kelly, Kathryn L. Burgio, Camille P. Vaughan

**Affiliations:** 1 Birmingham/Atlanta Geriatric Research, Education, and Clinical Center (GRECC), Department of Veterans Affairs, Birmingham, Alabama, and Atlanta, Georgia; 2 Department of Medicine, University of Alabama at Birmingham, Birmingham; 3 Now with Division of Geriatrics, Department of Internal Medicine, University of Utah, Salt Lake City; 4 Durham VA Health Care System, Durham, North Carolina; 5 Department of Medicine, Duke University School of Medicine, Durham, North Carolina; 6 Atlanta VA Health Care System, Atlanta, Georgia; 7 Nell Hodgson Woodruff School of Nursing, Emory University, Atlanta, Georgia; 8 Department of Medicine, Emory University, Atlanta, Georgia

## Abstract

**Importance:**

Initial treatment for urinary incontinence (UI) includes behavioral treatment. However, access to care may be limited.

**Objective:**

To compare the effectiveness of 2 remote modalities for UI behavioral treatments and to assess the effect of a booster video visit for treatment nonresponders.

**Design, Setting, and Participants:**

This sequential, multiple assignment randomized clinical trial was conducted in 3 southeastern US Department of Veterans Affairs health care systems from April 2020 through September 2023. Participants were nonpregnant women veterans with UI who had access to email.

**Interventions:**

Remote interventions included (1) a mobile health UI app (MyHealth*e*Bladder) with daily sessions delivered for 8 weeks or (2) a single video visit through VA Video Connect delivered by trained UI health care professionals and (3) a randomized booster video visit for women without UI symptom improvement at 8 weeks.

**Main Outcomes and Measures:**

The primary outcome was change in UI symptoms at 12 weeks as measured by the International Consultation on Incontinence Questionnaire–Urinary Incontinence Short Form (ICIQ-UI SF; score range, 0-21, with higher scores indicating more severe UI) with a minimal clinically important difference of 2.52 defining response status at 8 weeks. The results of the sequential addition of a video booster visit for nonresponders in both randomization groups were also investigated. Primary analyses were assessed using intention-to-treat and per-protocol analyses.

**Results:**

Of 286 women veterans randomized (147 to UI app; 139 to video visit), 244 (85%) received an intervention. Their mean (SD) age was 53.2 (11.3) years (range, 23-83 years). At 12 weeks, ICIQ-UI SF scores for the UI app group decreased −3.6 (95% CI, −4.4 to −2.8) points compared with −2.3 (95% CI, −3.1 to −1.5) points for the video visit group (*P* = .02). In the UI app nonresponder group at 8 weeks (n = 29 [20%]) with the booster video visit (n = 12), ICIQ-UI SF scores changed −1.0 (95% CI, −2.4 to 0.4) points vs −1.2 (95% CI, −1.3 to 3.7) points for 10 UI app nonresponders who continued UI app treatment (*P* = .10). In the video visit nonresponder group (n = 61 [44%]) with the booster video visit (n = 23), ICIQ-UI SF scores changed −0.9 (95% CI, −2.1 to 0.3) points vs −0.3 (95% CI, −1.9 to 1.2) points in 30 video visit nonresponders who continued treatment (*P* = .58). The change in ICIQ-UI SF scores for the UI app reached the MCID threshold (2.52 points) in 4 weeks (−2.9 [95% CI, −3.8 to −2.0] points), whereas the video visit group did not reach this threshold until 24 weeks (−3.4 [95% CI,−4.2 to −2.5] points).

**Conclusions and Relevance:**

This randomized clinical trial comparing 2 remote delivery modalities of behavioral UI treatment with randomized booster visits among women veterans found that UI symptoms improved earlier for the UI app intervention compared with the video visit intervention, without reaching a meaningful clinical between-group difference at 12 weeks. The addition of a booster video visit did not further improve UI symptoms. Further research could extend these modalities to broaden access to behavioral UI treatment.

**Trial Registration:**

ClinicalTrials.gov Identifier: NCT04237753

## Key Points

**Question:**

What is the comparative effectiveness of evidence-based urinary incontinence (UI) behavioral treatment with or without booster visits delivered through 2 remote modalities?

**Findings:**

In this randomized clinical trial of 286 women veterans with UI, participants who received behavioral UI treatment through a mobile health app reported earlier UI symptom reductions compared with through a video visit; however, the difference in symptom reductions between modalities was not clinically meaningful. Addition of a booster video visit did not further improve UI symptoms.

**Meaning:**

These findings suggested that receiving remote behavioral UI treatment through either a mobile health app or video visit improved UI symptoms among women veterans, without clinically meaningful differences between modalities.

## Teaser

This randomized clinical trial compares the effectiveness of urinary incontinence behavioral treatments delivered via a mobile health app vs a video visit and assesses the effect of a booster video visit for treatment nonresponders among women veterans.

## Introduction

Increasing numbers of women veterans are using the Department of Veterans Affairs (VA) for health care, including treatment for urinary incontinence (UI).^1,2^ Behavioral therapy (including pelvic floor muscle exercises, bladder control strategies, and fluid management) is effective in reducing UI and is a recommended treatment for UI.^3,4,5,6^ However, many VA Health Care Systems may not have trained health care professionals to educate patients and to administer these therapies. A review of quality of care for women veterans with new or worsening UI at 4 VA sites found that while close to 80% of patients had UI assessed, less than one-third received behavioral treatment.^7^

Behavioral UI treatment can be burdensome, especially for rural veterans, because it typically involves multiple in-person visits. According to the National Survey of Women Veterans, a self-reported reason for leaving VA care included distance to VA facilities.^8^ Telehealth technologies enable the provision of health care services that surmount the usual constraints of geographic distance, time, and social or cultural boundaries. The VA uses a specific platform, called VA Video Connect, to deliver remote telehealth care. Telehealth care in the VA has grown from 300 000 veterans served in 2010 with recent expansion reaching 2.3 million veterans in 2022; more than a third of all veterans have received some form of remote care.^9,10^ Women veterans are early adopters of VA video-based care delivery and often experience barriers to accessing clinical care.^11,12,13^

In prior work, a multidisciplinary team applied input from women veterans to design and pilot test an evidence-based 8-week behavioral mobile telehealth app for women veterans with UI.^14^ The UI app, MyHealth*e*Bladder, included pelvic floor muscle exercises, bladder control strategies, fluid management, risk factor reduction, self-monitoring, inspirational quotes, and story-based scenarios with women veterans managing UI. Other mobile health apps have efficacy for the remote delivery of UI treatment for women.^15^ However, this UI app is unique in its specificity to appeal to women veterans who have increased risk for UI and have shared military experiences.^16,17,18^

This study reports the main results of the Optimizing Remote Access to Urinary Incontinence Treatments for Women Veterans (PRACTICAL) trial, which used a sequential, multiple assignment, randomized trial (SMART) design to test the efficacy of remote delivery modalities for behavioral UI treatment, comparing the UI app to video visit, while also testing the addition of a video visit booster visit for women who did not report improved UI symptoms.^19^ The primary aim of the study was to determine the optimal remote modality. We hypothesized that remotely delivered behavioral therapy for UI via the UI app for 8 weeks would be superior to delivery through a video visit with a continence specialist. Our second aim used the SMART design with a second randomization to assess whether outcomes could be optimized at 12 weeks with the sequential addition of a video visit booster visit for women who did not respond with improved UI symptoms at 8 weeks.^20^

## Methods

### Design, Setting, and Participants

We compared the effectiveness of the UI app to video visits administered by a trained UI health care professional and evaluated the sequential addition of a video booster visit for nonresponders in both randomization groups. This SMART design included 2 randomization stages and compared 1 optimization factor at 8 weeks (defined as a booster video visit) for both groups during a 12-week intervention period, with UI severity assessed at baseline, 8 weeks, 12 weeks (primary outcome), and 24 weeks for durability.^14^ The trial protocol is provided in Supplement 1. The study was approved by the institutional review board at each VA site, and all women signed informed consent documents prior to randomization. This trial followed the Consolidated Standards of Reporting Trials (CONSORT) 2025 reporting guideline.^21^

Participants were nonpregnant women veterans (18 years or older) who met self-reported criteria for UI lasting at least 3 months and had access to email with a computer, tablet, or mobile phone. Women were excluded for new UI treatments started in the prior 3 months or for any planned UI treatments for the 24-week study duration, or for unstable medical conditions that could contribute to UI (ie, uncontrolled diabetes), unstable housing, neurologic conditions contributing to UI, genitourinary cancer, or less than 12 weeks post partum. No in-person evaluation for eligibility was required. The study provided financial incentives for completing research surveys ($25 each for a total of $100). A VA data and safety monitoring board provided annual monitoring.

### Randomization

We stratified eligible women according to VA site and by UI symptom severity using the same validated UI questionnaire used for outcome measurement. Within strata, participants were randomized remotely by a computer-generated algorithm and given access to start the UI app with email instruction (written and video) or given an appointment for the video visit through usual care clinical pathways. Research staff involved in data management were blinded to intervention assignments.

### Interventions

Participants received evidence-based behavioral treatment either through the UI app with daily sessions across 8 weeks or the same standardized content delivered through a single video visit or the booster video visit. The major components of the behavioral intervention for the UI app and the video visit have been published previously and include education on bladder anatomy and function, UI risk factor reduction, pelvic floor muscle exercises, bladder control strategies, and self-monitoring.^14,22,23^ The UI app also had stories and quotes to reinforce behavior change and acknowledge the experience of women veterans, as well as reminder features to promote adherence to the program. Women completed daily sessions with the UI app and had the opportunity to make up any missed sessions within 2 weeks from the assigned day. Both groups had access on the data collection platform to evidenced-based materials in booklet form adapted with pictures depicting women veterans.^19^

### Treatment Fidelity for the Video Visit

Centralized training was conducted to standardize content according to a protocolized treatment manual across all sites. Certified registered nurse practitioners who delivered UI care in Birmingham and Atlanta VA Health Care Systems implemented the video visit intervention for all sites (Atlanta, Georgia; Birmingham, Alabama; and Durham, North Carolina). The nurse practitioners completed standardized intervention sessions and fidelity notes immediately after each session. Supervision was provided by a study investigator at each site (A.D.M. and C.P.V.) via weekly meetings. These meetings included fidelity feedback, review of fidelity notes, and debriefing of individual sessions as needed.

### Measurement

#### Data Collection and Baseline Measures

All measures were entered by participants on the same platform for the UI app and video visit with email reminders. Baseline measures included self-reported demographic variables, including age, educational level, race and ethnicity, branch of military service, years in military service, height, and weight, and health-related data, including medical conditions (comorbidities and diabetes), prior UI medications, mental health conditions (depression, posttraumatic stress disorder), military sexual trauma, obstetrical history (parity and hysterectomy), and type of device used for the interventions.^24,25,26^ Race and ethnicity were assessed in this study because breakdown of race and ethnicity was required per funding reporting. Race categories were based on National Institutes of Health and VA reporting and included Black or African American, more than 1 race, White, or other, which included American Indian, Alaska Native, Asian, and Native Hawaiian or Other Pacific Islander. Ethnicity categories were Hispanic and not Hispanic.

#### Outcome Measures

The primary outcome measure was responses to the validated International Consultation on Incontinence Questionnaire–Urinary Incontinence Short Form (ICIQ-UI SF).^27,28,29^ The 3-item ICIQ-UI SF was completed at baseline, 8 weeks, and 12 weeks as the primary outcome time point, with durability completed at 24 weeks (Figure). Scores on the ICIQ-UI SF ranged from 0 to 21, with higher scores indicating greater UI severity. We determined responder or nonresponder status based on the ICIQ-UI SF responses at 8 weeks to enable a second randomization for the addition of a booster video visit. The ICIQ-UI SF minimal clinically important difference (MCID) score threshold (−2.52 points) defined a responder or a nonresponder.^29^ Secondary outcomes included overactive bladder measured by the ICIQ Overactive Bladder (OAB) score, which ranges from 0 to 16 based on a combination of 4 symptom severity scores, with higher scores indicating worse symptom severity,^27^ adherence to the pelvic floor muscle exercises,^30^ satisfaction and perceptions of treatment,^31^ and estimated improvement.^31^

**Figure.**
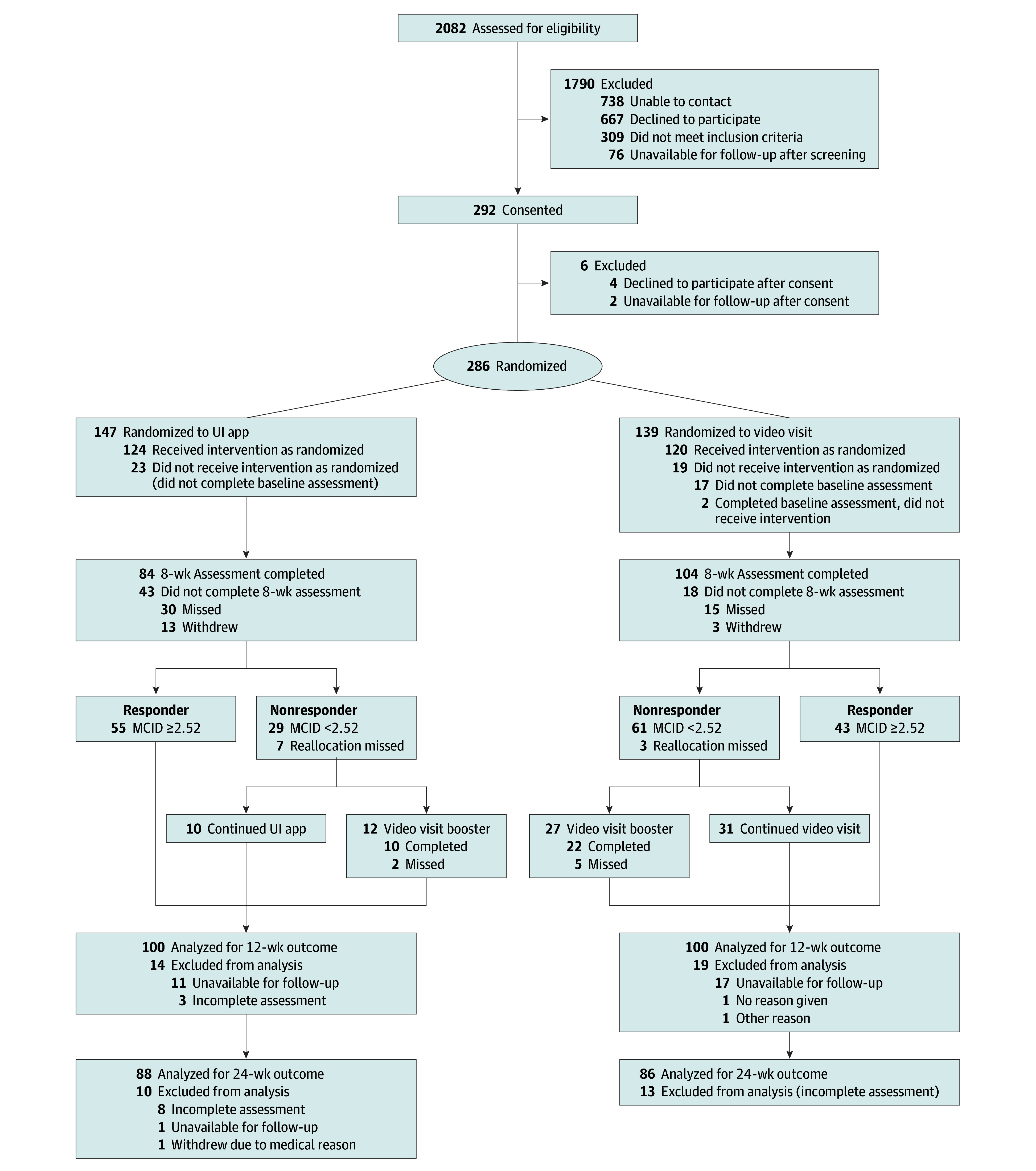
PRACTICAL Study CONSORT Diagram MCID indicates minimal clinically important difference; PRACTICAL, Optimizing Remote Access to Urinary Incontinence Treatments for Women Veterans trial; UI, urinary incontinence.

### Sample Size and Power Analysis

Sample size calculations for SMART designs have more than 1 power function.^32^ We powered this study for the first randomization stage and 8-week outcomes. Our pilot study had standardized effect size ranges from 0.40 to 0.45 for UI severity reduction at 8 weeks, resulting in projected total sample sizes ranging from 158 to 200 for 80% power at a 5% significance level. For the second randomization stage, we used the MCID of the ICIQ-UI SF (MCID, 2.52 ± 2.56) with 12-week outcomes.^28,33^ We assumed a 40% response rate (achievement of the MCID at 8 weeks), 30% in each arm would be rerandomized to the booster video visit or continued intervention, and thus, 70% of patients in each arm would remain on their initially randomized treatment program. With an initial sample size of 200 participants completing the first stage, 140 (70 in each arm) would be available for the 12-week end point comparison. This sample size yielded over 99% power to detect between-group differences at a 5% significance level based on the aforementioned UI severity reduction MCID at 12 weeks. To account for a 30% dropout rate, we recruited and retained 286 women for the baseline assessments.

### Statistical Analysis

Our primary analyses were intention to treat (ITT) and per protocol. Per-protocol analyses excluded participants without 8-week or 12-week scores and with less than 70% adherence to the UI app sessions or without video visits. All hypothesis tests were 2-sided, with *P* < .05 considered statistically significant. Baseline characteristics were summarized overall and by the comparison groups for the primary and secondary aims and tested by χ^2^ or Welch analysis of variance (ANOVA). To assess the primary aim (comparison of the UI app vs video visit), we performed repeated measures analyses using linear mixed models with heteroscedastic unstructured covariances. Follow-up testing compared groups on outcomes and change scores at each time point with Welch ANOVAs. A similar set of models were fit for comparing groups after the second randomization with the video visit booster. Normality assumptions were evaluated using residual plots and comparing results with tests performed using nonparametric procedures. Sensitivity analyses included covariate (eg, age, race and ethnicity, and VA site) adjusted models and modeling site as a random effect. Although mixed models are typically robust to missing data, we also addressed missing data for the primary aim using multiple imputation. All analyses were performed in SAS, version 9.4 (SAS Institute Inc).

## Results

### Baseline Characteristics

In total, 244 women veterans were randomized, completed baseline assessments, and received the intervention (Figure). Their mean (SD) age was 52.9 (11.3) years (range, 23-83 years), and 134 (55%) were Army veterans. Among these women, 142 (58%) self-identified as Black or African American, 6 (2%) as more than 1 race, 86 (35%) as White, 10 (4%) as other race, and 16 (6%) as Hispanic (Table 1). Women in both groups reported several comorbidities, including having mental health conditions (depression and posttraumatic stress disorder), being postmenopausal (156 [64%]), and 86 (35%) experiencing military sexual trauma. Greater percentages of women responders to initial treatment in the UI app group compared with the nonresponders reported being postmenopausal (45 [82%] vs 16 [55%], *P* = .009) and using a laptop or personal computer (39 [71%] vs 14 [48%], *P* = .04) for the intervention (eTable 1 in Supplement 2). Among women who received the initial intervention (Table 1), ICIQ-UI SF baseline scores were similar across intervention groups, without differences noted by UI severity or UI type. Dropout rates were high and similar in both groups (31% and 28% at 12 weeks; 38% and 41% at 24 weeks).

**Table 1.** Baseline Study Characteristics for Women Veterans Receiving Intervention and Completing Baseline Assessments, by Intervention Group

Characteristic	Participants, No. (%)	
	UI app	Video visit
No.	124	120
Age, mean (SD), y	53.1 (10.8)	53.4 (11.7)
Age, range, y	27-75	23-83
Ethnicity		
Hispanic	6 (5)	10 (8)
Not Hispanic	116 (93)	109 (91)
Not reported	2 (2)	1 (1)
Race		
Black or African American	80 (65)	62 (52)
More than 1	3 (2)	3 (2)
White	39 (31)	47 (39)
Other^a^	2 (2)	8 (7)
Educational level		
High school graduate	5 (4)	6 (5)
Some college	20 (16)	23 (19)
Associate degree	34 (27)	21 (17)
Bachelor’s degree	36 (29)	41 (34)
Graduate degree	29 (23)	29 (24)
Marital status		
Married	53 (43)	42 (35)
Divorced	37 (30)	43 (36)
Widowed	5 (4)	9 (7)
Separated	8 (7)	3 (2)
Never married	20 (16)	21 (17)
Unmarried couple	1 (1)	2 (2)
Branch of service		
Army	71 (57)	63 (52)
National Guard	3 (2)	4 (3)
Navy	16 (13)	17 (14)
Air Force	15 (1)	24 (20)
NOAA	0 (0)	1 (1)
Marine Corps	3 (2)	5 (4)
Coast Guard	2 (2)	1 (1)
Reserve	14 (11)	5 (4)
Years of service, mean (SD)	8.8 (7.7)	9.4 (8.0)
Median (IQR)	5.5 (4-11)	7 (3-15)
BMI, mean (SD)^b^	32.2 (6.4)	31.7 (6.9)
Median (IQR)	31 (28-36)	32 (26-37)
Self-reported characteristics		
Comorbidities, mean (SD)	8.1 (3.0)	7.6 (3.4)
Median (IQR)	8 (6-10)	7 (5-10)
Diabetes	14 (11)	17 (14)
Medication for UI	9 (7)	11 (9)
Depression	95 (77)	87 (72)
PTSD	67 (54)	55 (46)
Military sexual trauma	39 (31)	47 (39)
Hysterectomy	51 (41)	48 (40)
Postmenopausal	81 (65)	75 (63)
Parity		
None	11 (9)	21 (17)
1	23 (18)	21 (17)
2	34 (27)	32 (27)
3	29 (23)	24 (20)
≥4	27 (22)	22 (18)
Device type, not mutually exclusive		
Cell phone	100 (81)	98 (82)
Tablet	46 (37)	43 (36)
Laptop or PC	81 (65)	87 (72)
UI severity score, mean (SD)^c^	11.67 (4.53)	10.93 (3.91)
UI severity category^c^		
Mild (score 1-5)	8 (6)	10 (8)
Moderate (score 6-12)	67 (54)	63 (52)
Severe (score 13-21)	49 (39)	47 (39)
UI type		
Mixed	96 (79)	95 (79)
Urgency	11 (9)	14 (8)
Stress	12 (10)	10 (12)
Other	2 (2)	1 (1)

Abbreviations: BMI, body mass index; NOAA, National Oceanic and Atmospheric Administration; PC, personal computer; PTSD, posttraumatic stress disorder; UI, urinary incontinence.

^a^ Other included American Indian, Alaska Native, Asian, and Native Hawaiian or Other Pacific Islander.

^b^ BMI calculated as weight in kilograms divided by height in meters squared.

^c^ Scores based on responses to the International Consultation on Incontinence Questionnaire–Urinary Incontinence Short Form ranged from 0 to 21, with higher scores indicating greater UI severity.

### UI Outcomes

The ICIQ-UI SF completion rates for the 286 randomized women (mean [SD] age, 53.2 [11.3] years; range, 23-83 years) were 66% (n = 188) at 8 weeks, 70% (n = 200) at 12 weeks for the primary outcome, and 61% (n = 174) at 24 weeks. From the ITT analysis at 12 weeks (Table 2; eFigure in Supplement 2), ICIQ-UI SF scores for the UI app group changed by −3.6 (95% CI, −4.4 to −2.8) points vs −2.3 (95% CI, −3.1 to −1.5) points for the video visit group. The between-group difference in ICIQ-UI SF scores was −1.3 (95% CI, −2.4 to −0.2) points for the ITT analysis (*P* = .02) at 12 weeks. The change in ICIQ-UI SF scores for the UI app reached the MCID threshold (2.52 points) in 4 weeks (−2.9 [95% CI, −3.8 to −2.0] points), whereas the video visit group did not reach this threshold until 24 weeks (−3.4 [95% CI,−4.2 to −2.5] points) (Table 2; eFigure in Supplement 2).

**Table 2.** Intention-to-Treat and Per-Protocol Analyses of Between-Group Changes in ICIQ-UI SF Score at 12 Weeks and 24 Weeks^a^

Study visit	Total, No.	Analysis											
		Intention to treat						Per protocol					
		UI app		Video visit		Between-arm difference		UI app		Video visit		Between-arm difference^b^	
		No.	Mean (95% CI)	No.	Mean (95% CI)	Difference (95% CI)	*P* value	No.	Mean (95% CI)	No.	Mean (95% CI)	Difference (95% CI)	*P* value
Baseline	286	147	11.6 (10.9 to 12.3)	139	11.0 (10.3 to 11.6)	0.6 (−0.4 to 1.6)	.22	76	11.6 (10.5 to 12.7)	104	11.0 (10.3 to 11.7)	0.6 (−0.7 to 1.9)	.38
4 wk	187	85	8.7 (7.7 to 9.8)	102	9.7 (8.9 to 10.5)	−1.0 (−2.3 to 0.4)	.16	76	8.9 (7.7 to 10.1)	98	9.7 (8.9 to 10.5)	−0.8 (−2.2 to 0.6)	.27
4 wk Change from baseline	187	85	−2.9 (−3.8 to −2.0)	102	−1.3 (−2.0 to −0.6)	−1.6 (−2.8 to −0.5)	.004	76	−2.7 (−3.6 to −1.8)	98	−1.4 (−2.1 to −0.6)	−1.3 (−2.5 to −0.2)	.02
8 wk	188	84	8.3 (7.2 to 9.3)	104	9.0 (8.1 to 9.8)	−0.7 (−2.0 to 0.6)	.29	76	8.0 (6.9 to 9.1)	104	9.0 (8.1 to 9.8)	−0.9 (−2.3 to 0.4)	.17
8 wk Change from baseline	188	84	−3.4 (−4.4 to −2.4)	104	−2.0 (−2.7 to −1.3)	−1.4 (−2.6 to −0.2)	.03	76	−3.6 (−4.6 to −2.5)	104	−2.0 (−2.7 to −1.3)	−1.5 (−2.8 to −0.3)	.02
12 wk	200	100	8.3 (7.4 to 9.3)	100	8.8 (7.9 to 9.6)	−0.4 (−1.7 to 0.9)	.52	75	8.1 (6.9 to 9.2)	97	8.8 (7.9 to 9.7)	−0.7 (−2.2 to 0.7)	.33
12 wk Change from baseline (primary outcome)	200	100	−3.6 (−4.4 to −2.8)	100	−2.3 (−3.1 to −1.5)	−1.3 (−2.4 to −0.2)	.02	75	−3.5 (−4.5 to −2.6)	97	−2.3 (−3.2 to −1.5)	−1.2 (−2.5 to 0.0)	.05
24 wk	178	92	8.1 (7.0 to 9.2)	86	7.5 (6.6 to 8.5)	0.5 (−0.9 to 1.9)	.45	73	7.7 (6.4 to 9.0)	84	7.5 (6.5 to 8.4)	0.2 (−1.3 to 1.8)	.75
24 wk Change from baseline (secondary outcome)	178	92	−4.0 (−5.0 to −3.0)	86	−3.4 (−4.2 to −2.5)	−0.6 (−1.9 to 0.7)	.34	73	−4.1 (−5.3 to −2.9)	84	−3.5 (−4.3 to −2.6)	−0.6 (−2.1 to 0.8)	.39

Abbreviations: ICIQ-UI SF, International Consultation on Incontinence Questionnaire–Urinary Incontinence Short Form; UI, urinary incontinence.

^a^ ICIQ-UI SF scores ranged from 0 to 21, with higher scores indicating greater UI symptom severity.

From the per-protocol analysis—defined by receipt of an initial video visit, completion of 70% of the UI app sessions, and provision of self-report scores at 8 weeks and 12 weeks—(Table 2), ICIQ-UI SF scores for the UI app group at 12 weeks changed by −3.5 (95% CI, −4.5 to −2.6) points vs −2.3 (95% CL, −3.2 to −1.5) points for the video visit group. The between-group difference in ICIQ-UI SF scores was −1.2 (95% CI, −2.5 to 0.0) points for the per-protocol analysis (*P* = .05).

Continued improvements in ICIQ-UI SF scores were noted at 24 weeks for both groups. However, the differences between the groups in ICIQ-UI SF scores were not significant at 24 weeks in the ITT (−0.6 [95% CI, −1.9 to 0.7] points; *P* = .34) or per-protocol (−0.6 [95% CI, −2.1 to 0.8] points]; *P* = .34) analyses. Sensitivity analyses did not result in substantial differences in scores across groups (eTable 2 in Supplement 2).

In Table 3, women are further categorized by randomization group as responders and nonresponders per the SMART trial design. Women who were responders had higher baseline ICIQ-UI SF scores compared with the nonresponders both in the UI app group (12.5 [95% CI, 11.3-13.7] points vs 10.0 [95% CI, 8.3-11.8] points; *P* = .02) and in the video visit group (12.1 [95% CI, 11.1-13.2] points vs 10.2 [95% CI, 9.2-11.2] points; *P* = .009). In the UI app nonresponder group (n = 29 [20%]) who had a booster video visit (n = 12), ICIQ-UI SF scores changed −1.0 (95% CI, −2.4 to 0.4) points compared with 1.2 (95% CI, −1.3 to 3.7) points in UI app nonresponders who continued treatment (n = 10) (*P* = .10). In the video visit nonresponder group (61 [44%]) with the booster video visit (n = 23), ICIQ-UI SF scores changed −0.9 (95% CI, −2.1 to 0.3) points compared with −0.3 (95% CI, −1.9 to 1.2) points in the video visit nonresponders group who continued treatment (n = 30) (*P* = .58).

**Table 3.** Comparisons of ICIQ-UI SF Scores for Responders and Nonresponders by Randomization Group

Study visit	UI app (n = 84)						Video visit (n = 104)					
	UI app			Nonresponders second randomization (n = 22 of 29)			Video visit			Nonresponders second randomization (n = 58 of 61)		
	Responders, mean (95% CI)	Nonresponders, mean (95% CI)	*P* value	UI app continue, mean (95% CI)	UI app with video booster, mean (95% CI)	*P* value	Responders, mean (95% CI)	Nonresponders, mean (95% CI)	*P* value	Video visit continued, mean (95% CI)	Video visit with video booster, mean (95% CI)	*P* value
No.	55	29		10	12		43	61		31	27	
Baseline	12.5 (11.3 to 13.7)	10.0 (8.3 to 11.8)	.02	10.0 (6.8 to 13.2)	9.3 (6.1 to 12.4)	.71	12.1 (11.1 to 13.2)	10.2 (9.2 to 11.2)	.009	10.6 (9.2 to 12.0)	9.6 (8.0 to 11.1)	.32
4 wk^a^	8.0 (6.8 to 9.2)	10.6 (8.2 to 13.0)	.06	11.4 (6.5 to 16.4)	9.3 (5.7 to 12.9)	.44	8.4 (7.4 to 9.5)	10.5 (9.4 to 11.6)	.008	11.2 (9.7 to 12.8)	9.4 (7.7 to 11.1)	.12
4 wk Change from baseline	−4.5 (−5.4 to −3.5)	0.7 (−0.5 to 1.9)	<.001	1.7 (−0.5 to 3.9)	0.1 (−1.7 to 1.9)	.23	−3.9 (−5.0 to −2.8)	0.3 (−0.3 to 1.0)	<.001	0.6 (−0.3 to 1.6)	0.0 (−0.9 to 0.9)	.33
8 wk	6.6 (5.6 to 7.6)	11.4 (9.6 to 13.3)	<.001	11.9 (8.5 to 15.3)	9.4 (6.1 to 12.7)	.25	6.7 (5.8 to 7.7)	10.5 (9.4 to 11.6)	<.001	10.9 (9.5 to 12.3)	9.6 (7.8 to 11.4)	.25
8 wk Change from baseline	−5.9 (−6.7 to −5.1)	1.4 (0.5 to 2.2)	<.001	1.9 (0.2 to 3.6)	0.2 (−0.9 to 1.2)	.08	−5.4 (−6.1 to −4.6)	0.3 (−0.2 to 0.9)	<.001	0.3 (−0.5 to 1.1)	0.0 (−0.7 to 0.8)	.60
12 wk^b^	7.4 (6.1 to 8.6)	9.8 (7.9 to 11.8)	.03	11.2 (7.8 to 14.6)	8.3 (4.8 to 11.7)	.19	7.3 (6.2 to 8.4)	9.9 (8.6 to 11.2)	.003	10.1 (8.2 to 12.1)	9.0 (7.1 to 11.0)	.42
12 wk Change from baseline (primary outcome)	−5.2 (−6.1 to −4.4)	−0.2 (−1.3 to 0.9)	<.001	1.2 (−1.3 to 3.7)	−1.0 (−2.4 to 0.4)	.10	−4.9 (−5.9 to −3.9)	−0.4 (−1.4 to 0.5)	<.001	−0.3 (−1.9 to 1.2)	−0.9 (−2.1 to 0.3)	.58
24 wk^c^	6.7 (5.4 to 8.1)	10.3 (8.0 to 12.5)	.008	10.7 (6.7 to 14.7)	8.9 (4.5 to 13.3)	.51	7.0 (5.7 to 8.2)	7.9 (6.5 to 9.3)	.33	8.5 (6.6 to 10.4)	6.6 (4.4 to 8.9)	.20
24 wk Change from baseline	−6.0 (−7.2 to −4.8)	0.0 (−1.4 to 1.5)	<.001	0.7 (−2.6 to 4.0)	−0.7 (−3.0 to 1.5)	.43	−5.4 (−6.6 to −4.1)	−1.9 (−2.9 to −0.9)	<.001	−1.6 (−3.0 to −0.2)	−2.5 (−4.2 to −0.8)	.39

Abbreviations: ICIQ-UI SF, International Consultation on Incontinence Questionnaire–Urinary Incontinence Short Form; UI, urinary incontinence.

^a^ At 4 weeks, UI app responders, n = 51 (4 missing); UI app nonresponders, n = 26 (3 missing); video visit responders, n = 39 (4 missing); video visit nonresponders, n = 59 (2 missing). At 4 weeks, UI app continued, n = 9 (1 missing). At 4 weeks, video visit and video booster, n = 26 (1 missing).

^b^ At 12 weeks, UI app responders, n = 54 (1 missing); UI app nonresponders, n = 29 (0 missing); video visit responders, n = 41 (2 missing); video visit nonresponders, n = 56 (5 missing). At 12 weeks, video visit continued, n = 30 (1 missing). At 12 weeks, video visit and video booster, n = 23 (4 missing).

^c^ At 24 weeks, UI app responders, n = 50 (5 missing); UI app nonresponders, n = 28 (1 missing); video visit responders, n = 38 (5 missing); video visit nonresponders, n = 46 (15 missing). At 24 weeks, UI app and video visit, n = 11 (1 missing). At 24 weeks, video visit continued, n = 26 (5 missing). At 24 weeks, video visit and video booster, n = 18 (9 missing).

### Secondary Outcomes

In the ITT analysis (Table 4), women in the UI app group reported greater improvements in OAB symptom severity scores at 12 weeks (−2.5 [95% CI, −3.0 to −2.0] points) compared with the video visit group (−1.8 [95% CI, −2.3 to −1.3) points; P = .06), and were more likely to achieve improvements reflecting the MCID for the ICIQ-UI SF. Similarly, in the per-protocol analysis, women in the UI app group reported greater improvements in OAB symptom severity scores at 12 weeks (−2.8 [95% CI, −3.4 to −2.2]) compared with the video visit group (−1.8 [95% CI, −2.2 to −1.3]; P = .007). At 24 weeks, there were no differences between the groups in OAB symptom severity scores in either the ITT or the per-protocol analysis. In total, 90% of users of the UI app and video visit reported having satisfaction at 12 weeks. Adherence rates to pelvic floor muscle exercises were at 90% or more at 12 weeks, without differences between groups. Similarly, there were no group differences in the miles saved by not having to travel for an in-person visit. There were no clinically meaningful adverse events reported throughout the trial.

**Table 4.** Intention-to-Treat and Per-Protocol Analyses of Between-Group Changes in ICIQ-OAB Scores and Other Secondary Outcomes

Secondary outcome	Total No.	Analysis											
		Intention to treat						Per protocol					
		UI app		Video visit		Between-arm difference		UI app		Video visit		Between-arm difference	
		No.	Mean (95% CI)	No.	Mean (95% CI)	Difference (95% CI)	*P* value	No.	Mean (95% CI)	No.	Mean (95% CI)	Difference (95% CI)	*P* value
ICIQ-OAB scores^a^													
Baseline	247	125	7.8 (7.2 to 8.4)	122	7.6 (7.0 to 8.1)	0.2 (−0.6 to 1.0)	.60	75	8.1 (7.3 to 8.8)	97	7.7 (7.2 to 8.3)	0.3 (−0.7 to 1.3)	.53
12 wk	199	99	5.4 (4.8 to 6.0)	100	5.9 (5.2 to 6.6)	−0.5 (−1.5 to 0.4)	.25	75	5.2 (4.5 to 6.0)	97	6.0 (5.3 to 6.7)	−0.7 (−1.8 to 0.3)	.14
Change from baseline	199	99	−2.5 (−3.0 to −2.0)	100	−1.8 (−2.3 to −1.3)	−0.7 (−1.4 to 0.0)	.06	75	−2.8 (−3.4 to −2.2)	97	−1.8 (−2.2 to −1.3)	−1.1 (−1.8 to −0.3)	.007
24 wk	177	91	5.5 (4.9 to 6.2)	86	5.3 (4.7 to 6.0)	0.2 (−0.7 to 1.1)	.69	72	5.4 (4.7 to 6.2)	84	5.3 (4.6 to 6.0)	0.1 (−0.9 to 1.2)	.79
Change from baseline	NA	91	−2.4 (−3.0 to −1.9)	86	−2.2 (−2.7 to −1.6)	−0.3 (−1.0 to 0.5)	.52	72	−2.6 (−3.3 to −1.9)	84	−2.2 (−2.7 to −1.7)	−0.4 (−1.3 to 0.5)	.36
Adherence to pelvic floor muscle exercises													
4 wk	188	87	82 (94.3) [89.4 to 99.1]	101	98 (97.0%) [93.7 to 100]	−2.8 (−8.7 to 3.1)	.35	76	71 (93.4) [87.9 to 99.0]	97	94 (96.9) [93.5 to 100]	−3.5 (−10 to 0-3.1)	.28
8 wk	189	85	77 (90.6) [84.4 to 96.8]	104	101 (97.1%) [93.9 to 100]	−6.5 (−13.5 to 0.5)	.06	76	69 (90.8) [84.3 to 97.3]	104	101 (97.1) [93.9 to 100]	−6.3 (−13.6 to 0.9)	.07
12 wk	196	98	89 (90.8) [85.1 to 96.5]	98	91 (92.9%) [87.8 to 98.0]	−2.0 (−9.7 to 5.6)	.60	75	69 (92.0) [85.9 to 98.1]	95	88 (92.6) [87.4 to 97.9]	−0.6 (−8.7 to 7.4)	.88
24 wk	176	90	78 (86.7) [79.6 to 93.7]	86	77 (89.5) [83.1 to 96.0]	−2.9 (−12.4 to 6.7)	.56	71	62 (87.3) [79.6 to 95.1]	84	75 (89.3) [82.7 to 95.9]	−2.0 (−12.1 to 8.2)	.70
Satisfaction, perceptions of improvement at 12 wk													
Satisfaction (PSQ), mean (95% CI)^b^	193	98	1.4 (1.3 to 1.5)	95	1.3 (1.2 to 1.4)	0.1 (−0.1 to 0.2)	.46	75	1.3 (1.2 to 1.5)	92	1.3 (1.2 to 1.4)	0.0 (−0.2 to 0.2)	.96
PSQ, dichotomized^b^	193	98	91 (92.9)	95	92 (96.8)	−4.0 (−10.1 to 2.2)	.21	75	70 (93.3%)	92	89 (96.7)	−3.4 (−10.1 to 3.3)	.31
GPI^c^	194	98	1.8 (1.6 to 2.0)	96	2.0 (1.8 to 2.1)	−0.2 (−0.4 to 0.1)	.15	75	1.7 (1.5 to 1.9)	93	1.9 (1.8 to 2.1)	−0.2 (−0.5 to 0.0)	.05
GPI, dichotomized^c^	194	98	80 (81.6)	96	76 (79.2)	2.5 (−8.7 to 13.6)	.67	75	64 (85.3)	93	74 (79.6)	5.8 (−5.7 to 17.2)	.33
Estimated percent improvement^d^	193	97	68.3 (62.4 to 74.2)	96	61.4 (55.4 to 67.4)	6.9 (−1.4 to 15.3)	.11	77	70.2 (63.4 to 77.1)	93	62.1 (56.0 to 68.2)	8.1 (−1.0 to 17.3)	.08
Miles saved per visit													
Mean (95% CI)	285	146	25.6 (21.3 to 29.8)	139	29.9 (25.1 to 34.7)	−4.3 (−10.7 to 2.1)	.19	75	26.0 (19.4 to 32.5)	97	27.2 (22.1 to 32.4)	−1.3 (−9.5 to 7.0)	.76
Median (IQR)	285	285	16 (10 to 30)	139	23 (12 to 36)	NA	.07	75	18 (10 to 30)	97	21 (11 to 32)	NA	.42

Abbreviations: GPI, global perceptions of improvement; ICIQ-OAB, International Consultation on Incontinence Questionnaire–Overactive Bladder; NA, not available; PSQ, Patient Satisfaction Questionnaire; UI, urinary incontinence.

^a^ ICIQ-OAB score ranges from 0 to 16 based on a combination of 4 symptom severity scores (0-4 per symptom for daytime frequency, nocturia, urgency, and incontinence), with higher scores indicating worse symptom severity.

^b^ Global ratings of Patient Satisfaction and Improvement Questionnaire (3 questions): PSQ dichotomized as completely or somewhat vs not at all, with scores ranging from 0 to 2 and higher values indicating more satisfaction across categories; global rating dichotomized as much better or better vs about the same, worse, or much worse.

^c^ GPI score ranges from 0 to 4, with higher scores representing more improvement across categories; global rating dichotomized as much better or better vs about the same, worse, or much worse.

^d^ Estimated percent improvement ranged from 0% to 100%, with higher scores indicating greater improvement.

## Discussion

In this randomized clinical trial using a SMART design, we determined that virtual modalities are effective for the remote delivery of behavioral UI treatment for women veterans. Women veterans reported improved UI symptoms with both remote delivery modalities, with earlier UI symptom improvement reported among women randomized to a mobile health app, compared with a video visit. Optimization of UI treatment with a booster video after 8 weeks did not further improve UI symptoms for nonresponders in either randomization group. Although women had earlier improvements in UI severity scores with the UI app than the video visit, the between-group differences did not reach clinically important differences at 12 weeks.

A recent systematic review highlights the potential for remote delivery of pelvic floor muscle–based behavioral therapy via video telehealth for UI, with small previous trials showing comparative efficacy of remote delivery of treatment with in-person delivery.^34^ Additionally, previous studies of mobile health apps for the treatment of UI among women have highlighted their potential utility compared with usual care.^35^ The PRACTICAL trial was unique in comparing 2 remote delivery methods for UI treatment among women using a SMART design, with the goal of informing best practices for broader dissemination within the VA Healthcare system and beyond. Compared with the results from Asklund et al,^33^ which evaluated the Swedish Tät app to a waitlist control, similar reductions in UI frequency were observed among participants in the PRACTICAL UI app group, reinforcing the potential of self-directed UI treatment through a mobile health app.

The PRACTICAL trial was important for demonstrating the generalizability of the app, which was designed in partnership with women veterans. The PRACTICAL study included women reflective of veterans enrolled in the VA Healthcare System in the Southeastern US. Through a prior pilot study and a detailed methodology publication, the UI app content was curated to ensure stories and images of women veterans highlighted shared experiences and inspirational quotes for motivation.^14,19^ Notably, more than half of the population self-identified as Black or African American, and most women were over 50 years of age. Many users of the UI app reported accessing modules through multiple types of devices, including a smartphone-based app and a laptop or personal computer through a web-based module.

### Limitations

Our study has limitations that include the low UI response rate for nonresponders who were offered a video booster session, differing nonresponder rates across the groups, as well as not reaching a MCID difference in our primary outcome between the interventions. Although the video booster session was meant to optimize treatment through additional consultation with a continence specialist, negligible UI improvement was reported. While the sample is too small for definitive analysis of women who were rerandomized, the results suggested that those who do not respond to a remote delivery approach should likely have further evaluation and may need an in-person assessment. Interestingly, women who did not respond to treatment also had less severe UI symptoms at baseline compared with the women who responded to treatment. Although we did not expect this finding, we anticipated that 30% of all women would not initially respond to treatment. We also found that a higher proportion of women in the video visit group compared with the UI app group were deemed nonresponders. While statistical significance was attenuated at 12 weeks in the per-protocol analysis compared with the ITT analysis, women assigned to the UI app and video visit reported similar reductions in UI symptoms without clinically significant between-group differences. Additionally, in the ITT analyses, the results suggested that women veterans assigned to the UI app achieved clinically meaningful reduction in UI symptoms earlier than those assigned to video visit. Dropout rates were high in both groups (31% and 28% at 12 weeks; 38% and 41% at 24 weeks) and consistent with our sample size calculations.

## Conclusions

In this randomized clinical trial, we provided evidence that remote delivery of behavioral UI treatment with a mobile health app compared with a video visit had earlier UI symptom improvement without differences in predefined clinical thresholds of UI symptom improvement between groups. Further research could extend these modalities to broaden access to behavioral UI treatment across the VA health care system and in other health care systems.

## Notes

Supplement 1.
Trial Protocol

Supplement 2. **eTable 1.** Baseline Descriptive Data for Responders and Non-Responders at 8-Weeks **eTable 2.** Sensitivity Analyses: Multiple Imputation and Linear Mixed Model Analyses of Between Group Changes in ICIQ-UI Scores **eFigure.** ICIQ-UI SF Score Change by Visit

Supplement 3.
Data Sharing Statement
