# Supplementary material for: Remote Access to Urinary Incontinence Treatments for Women Veterans: The PRACTICAL Randomized Clinical Trial
Source: JAMA Netw Open. 2025 Sep 16;8(9):e2532111. doi: 10.1001/jamanetworkopen.2025.32111 (PMC12441875; doi:10.1001/jamanetworkopen.2025.32111)
Supplement: Supplement 1. — Trial Protocol [file jamanetwopen-e2532111-s001.pdf]

2022

Optimizing Remote Access to Urinary Incontinence Treatment for Women Veterans: PRACTICAL

BIRMINGHAM PI: ALAYNE MARKLAND, ATLANTA PI: CAMILLE VAUGHAN, DURHAM PI: KAREN GOLDSTEIN

Version Date: 11/13/2022

I. Background and Significance:

Primary care and prevention research among women Veterans across the life cycle is an area of increasing focus within VA Health Administration (VHA).[1] Increasing numbers of women Veterans are using the VA for their general and gender-specific health care, representing a doubling in the past decade with 7% of all Veterans seen in the VHA being women. The number of women Veterans aged 50 and older is increasing within the VHA.[2] Women in this age range suffer from increased rates of urinary incontinence (UI).[3] Many women Veterans experience UI and may be at increased risk due to exposures during military service, such as restricted toilet access and the impact of heavy protective gear and equipment on the pelvic floor. These factors, along with known risk factors such as pregnancy, childbirth, and menopausal transitions increase UI risk among women Veterans. A recent study showed that 20 percent of women Veterans had UI.[4,5] The association of UI with post-traumatic stress and mood disorders in women Veterans returning from active service is profound and UI rates increase among women Veterans with post-traumatic stress disorder (PTSD), anxiety, and lifetime sexual assault.[4,5]

This proposed study focuses on improving access to first-line treatments for women Veterans with UI. Specifically, behavioral self-management treatments, including pelvic floor muscle training (PFMT), bladder control and voiding strategies, and fluid management, are widely recommended by consensus groups and guidelines as first-line treatment options because of their demonstrated effectiveness and low risk of side effects.[6] Persons implementing behavioral therapy for UI report greater confidence in self-management when provided with input from a clinician (physician, advanced practice provider, rehabilitation specialist) with specific training in UI management.[7] To identify gaps in UI treatment modalities within VHA, our group developed discipline-specific, web-based surveys to query VA providers about the care available for women Veterans with UI at VA Medical Centers (VAMCs) across the nation. After surveying gynecologists, urologists, and behavioral and physical therapy providers for first-line treatment options, we found that only 55% of facilities reported offering pelvic floor muscle training (PFMT), while 14% referred to another VA, and 44% referred to non-VA care. Sixteen percent of facilities had no way to provide PFMT. Our data demonstrate that women Veterans have limited access to clinicians who can provide these safe and effective treatments.

Given the identified need to improve access to first-line treatments for UI, we developed and tested two remote telehealth delivery methods: (1) a web-based educational platform called **MyHealtheBladder** and will compare to (2) video visits on a mobile device or desktop computer delivered via **VA Video Connect (VVC)** direct to women Veterans in their location of choice. We demonstrated the feasibility of these two models with funding from the Women Veterans Strategic Healthcare Group, Office of Patient Care Services and with funding from the Office of Geriatrics and Extended Care. In response to HSR&D research priority domains of improving access, the proposed study will incorporate a sequential, multiple assignment, randomized trial (SMART) design to determine the optimal method for remote delivery of behavioral therapy for UI to women Veterans. Additionally, we propose to use qualitative methods to identify key factors influencing participation in treatment through both models.

Upon successful completion of this proposal to examine the effectiveness of our tested remote telehealth delivery methods, MyHealtheBladder compared to VVC, we expect to have further evidence to support improved remote access for first-line UI treatments tailored for women Veterans. The results will be far-reaching, because UI is highly prevalent in women (up to 50% of women experience mild forms of UI), access to treatment is often unavailable, and if treated early, UI may not progress to more severe symptoms. The VA presents a unique opportunity to enhance health care management of UI remotely given the existing infrastructure for telehealth and programs to reach rural Veterans. The Women's Health Practice-Based Research Network (WH-PBRN) also represents a unique opportunity to further study the most effective delivery modality across a wide array of facilities, thus increasing generalizability and quality of care.[36] The intended audience for this research is women Veterans and their primary care providers. This proposal is responsive to the updated 2018 HSR&D and VHA ORD Priority areas, Access to Care, Women's Health, Virtual Care/Telehealth, Disability, Implementation Science with this Hybrid Effectiveness-Implementation Design Type I, as well as to increase substantial real-world impact of VA research for UI treatments.

II. Specific Aims and Hypotheses:

The *broad goal* is to improve access to evidenced-based first-line UI treatment for women Veterans using the most effective remote delivery modality for improving UI symptoms. Our multi-disciplinary team, including expertise from the

Women's Health Practice Based Research Network (WH-PBRN), the Birmingham/Atlanta Geriatrics Research, Education, and Clinical Center (GRECC), and the Durham Center of Innovation (COIN), is uniquely positioned to complete this proposal with the following specific aims:

Aim 1: Compare the effectiveness among women Veterans of two remote delivery models of evidenced-based UI treatment using MyHealtheBladder versus VA Video Connect (VVC).

Hypothesis 1: Women Veterans who receive the MyHealtheBladder interactive intervention will achieve greater reduction in UI severity compared to a remote VVC visit.

Aim 2: Optimize UI symptom improvement for women who do not respond to each type of remote delivery.

Hypothesis 2: Women Veterans who do not respond sufficiently to each intervention will have decreased UI severity with an additional remote VVC visit.

Aim 3: Explore women Veterans' and providers' perceptions of key factors that may influence future remote UI treatment dissemination across the VHA.

Hypothesis 3: Using interviews with women Veterans and providers, we will identify key concepts related to both modes of delivery that will inform future refinement and effectiveness more broadly across the VHA.

III. Research Design and Methods:

Overview: Members of our team have developed and pilot-tested two remote delivery methods for women Veterans with UI: MyHealtheBladder delivered via TONIC and video visits delivered via VA Video Connect. TONIC is a web-based educational platform that features the interactive intervention of MyHealtheBladder. MyHealtheBladder has branching logic to treat the most bothersome UI symptoms, including both stress and urgency UI. In order to compare the most effective remote delivery methods for women Veterans with UI, we will randomize women Veterans to MyHealtheBladder and compare the program to a 1:1 VA Video Connect (VVC) visit performed by UI experts in Birmingham and Atlanta. This trial uses a sequential, multiple assignments randomized trial or SMART design to explore the need to do 1:1 VVC visits for non-responders. The major features of the behavioral intervention for the MyHealtheBladder and the 1:1 VVC visit includes: education on the bladder and pelvic floor (bladder and pelvic floor muscle anatomy and function), self-monitoring measures (bladder diaries, adherence to pelvic floor muscle exercises), and evidenced-based core UI treatment components (pelvic floor muscle exercise instruction and bladder control strategies).

Figure 4. Remote UI Telehealth Treatment Trial Design

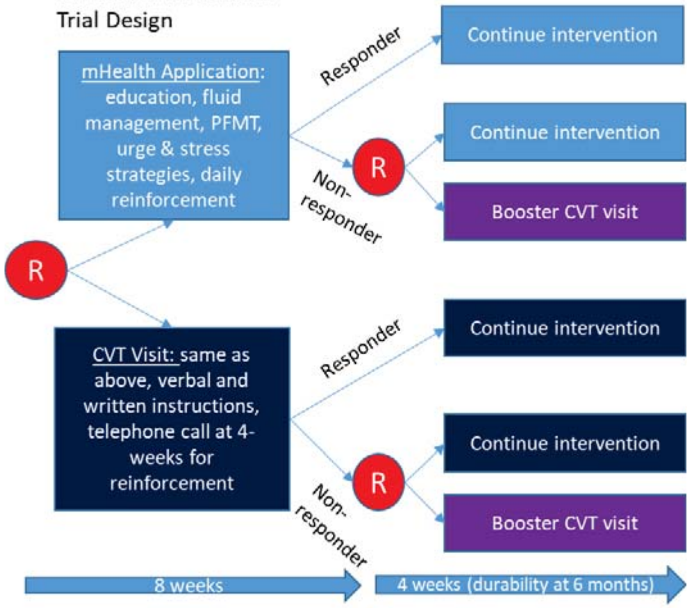

With this proposal, we aim to compare the effectiveness of the previously-tested MyHealtheBladder to a VVC visit administered by UI clinical providers (best usual care) by evaluating the sequential addition of a 1:1 VVC booster visit for non-responders in both randomization groups. This SMART design (Figure 1) will include two randomization stages and compare 1 optimization factor (VVC visit) over a 3-month intervention period with outcomes assessed at baseline, 8-weeks, 12-weeks (primary outcome), and 24-weeks (durability). A validated UI severity index (3 items ICIQ-UI) will determine a responder or non-responder to the program with a defined minimal clinically important difference (MCID) used as our responder/non-responder threshold. With the SMART design, we will investigate the effectiveness of two remote methods for the clinical delivery

of a behavioral self-management program that reduces UI severity and improves access to care for women Veterans (n=286) at 3 clinical sites: Birmingham, Atlanta, and Durham.

This design answers two key questions: 1) Can we improve remote access to evidenced-based behavioral UI treatments for women Veterans via MyHealtheBladder over a single VVC visit? and 2) Is a 1:1 visit necessary to optimize treatment?

Participants: We will recruit female Veterans (n=286) without any age limits who meet diagnostic criteria for UI. See Table 1 for inclusion/exclusion criteria. Based on screening and attrition rates in prior trials and pilot studies conducted at our sites, we anticipate screening 60% more participants (2:1 screening rate) to achieve the intended sample size. Women may be randomized if prior behavioral therapy (given at least 6 months prior to participation) was ineffective or the women report poor adherence to prior treatments.

Table 1: Inclusion and Exclusion Criteria
**Inclusion criteria:**
• Women Veterans
• Urinary incontinence occurring at least monthly for 3 months
• Able to access daily internet via computer or mobile device
• Access to personal email for MyHealtheBladder and VVC visit initiation and reminders
**Exclusion criteria:**
• Unstable medical conditions that could contribute to incontinence (e.g., recent major hospitalization, planned major surgery, conditions that affect urine volume - hemoglobin A1c of ≥ 9.0, chronic kidney disease with planned dialysis within 3 months, as assessed by PI or Site PI)
• Unstable psychiatric conditions (e.g., psychosis, suicidal, active alcohol/substance abuse based on history and medical records)
• Unstable housing situation
• Genitourinary cancer undergoing active treatment with chemotherapy or radiation
• Neurologic conditions known to contribute to incontinence (Multiple Sclerosis, Parkinson's Disease, TBI, Dementia, and Stroke Survivors with limited mobility)
• New treatments for incontinence started in the prior 3 months or planned during the 6-month study duration, includes medications and/or surgery
• < 12 weeks postpartum (including women currently pregnant)
• Current or prior surgically implanted sacral nerve stimulation device or botulinum toxin bladder injections for UI

Participant Recruitment: Based on screening and attrition rates in prior trials and pilot studies conducted at our sites, we anticipate screening 60% more participants (2:1 screening rate) to achieve the intended sample size (n=286). Women Veterans will be identified through 3 sources: 1) A letter mailed once at the beginning of the study and the end of years 1-3 to women who have received care at the three participating sites, including surrounding community-based outpatient clinics (CBOCs). The data on these women Veterans will be obtained remotely with assistance from the Women's Health Practice-based Research Network to help facilitate recruitment at each site. Lists of women Veterans will be provided to the study team at each site for the mailed letter. The letter will inform women Veterans about the study; 2) referrals from clinic providers who are included as co-Investigators on this proposal; and 3) targeted recruitment informational sessions at each site; and 4) flyers/advertisements posted in the medical centers and on VA-approved social media websites and applications. Women who respond will undergo a telephone screening assessment by trained research staff at each site. We will track monthly the number of screening telephone calls completed at each site. Individuals who meet initial screening criteria will be informed about the study and asked to provide written consent by mail or via the internet (or in-person, if desired). Numbers of consents obtained will be tracked monthly. No in-person evaluation for eligibility will be required for this study. However, if a woman Veteran desires in-person evaluation prior to randomization, the research team will coordinate a visit to the women's health clinic or gynecologic clinic at each site. After initial screening, in order to minimize exclusions and be consistent with our pilot data, women who received prior treatment for UI will be eligible if current treatments continue (e.g. medication) and surgery will not be planned during the 6-month assessment period. The baseline demographics and medical history will be obtained over the phone. If a woman has diabetes, we may recommend repeat A1C testing, if not performed in the last 6 months, to assess hyperglycemia, which can increase urine volume and worsen UI. Each participant's medical record will be reviewed for exclusion criteria (e.g., uncontrolled diabetes).

Data for Recruitment and Enrollment: Based on published cohort data (n=968), 20% of women Veterans (mean age of 38.7±8.7 years) reported UI.[5] Thus, we expect that at least 20% of the women Veterans at each site will be eligible for participation, for Atlanta (n=2,492) and for Durham (n=1,217). In the event that only 25% of the women Veterans with UI would be eligible or willing to participate (25% in Atlanta is 623 and Durham 304), we would still anticipate having the ability to recruit women for our proposed sample size of up to 110 women Veterans per site (n=286 total with a 25% drop out rate). Sites will continue to enroll until the end of the recruitment period or when the goal is met, whichever occurs first. The Birmingham and Durham sites may exceed the original protocol's goal of 86-87 women enrolled, per site, to account for drops and/or another site's under-recruitment.

Study Design (Figure 2):

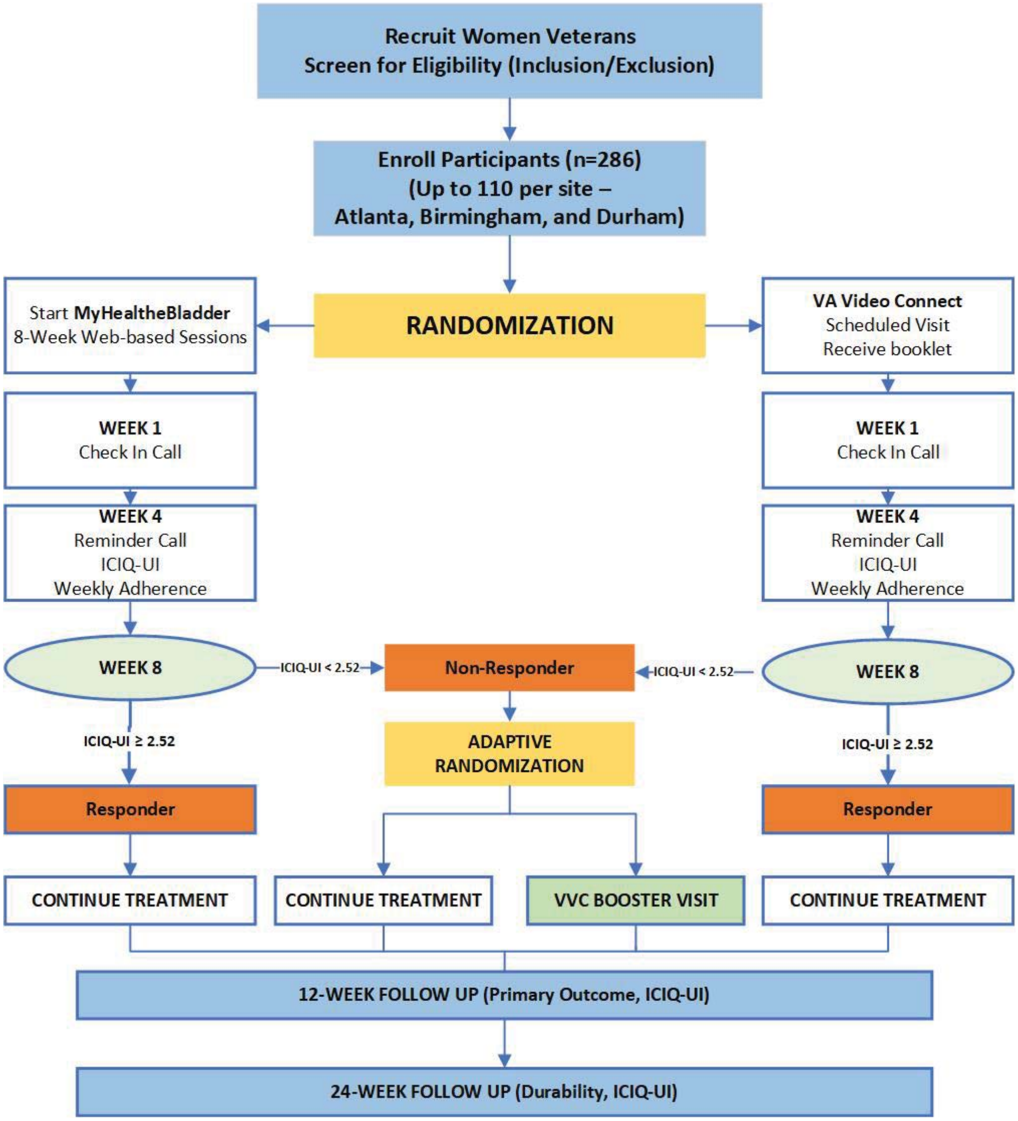

Primary Outcome, Secondary Outcomes, and Key Variables (Table 2, Figure 2): For this study, we used the validated International Consultation on Incontinence Questionnaire – Urinary Incontinence Short Form (**ICIQ-UI SF**) for the **primary outcome measure** based on psychometric properties and use in other studies using similar interventions.[42,46-48] Specifically, we powered this study and the sample size using the minimally important clinical difference (ICIQ-UI Short Form, Range is 0-21 and MICD is 2.52 (SD 2.56)) based on a Swedish study that had improvements in stress UI with an internet-based and mobile application-based pelvic floor muscle training intervention.[42] In this study (n=123), the

authors reported improvements with a mean **ICIQ-UI** SF score of 11.1 (SD 2.8) at baseline and a mean **ICIQ-UI** SF score reduction of 3.9, 95% confidence interval 3.0-4.7 after 3 months.[33]

Secondary outcomes include other lower urinary tract symptoms, impact on quality of life, adherence to the pelvic floor muscle exercises, satisfaction with treatment, adaptive behaviors, cost measures, and a technology usability questionnaire. **Key variables for covariates** using existing common elements include demographic variables, such as age, education, marital status, and race/ethnicity, including military sexual trauma. Other factors include medical and mental health conditions, perceived stress, key medications related to bladder symptoms, and sleep quality.

Enrollment and Randomization: No in-person assessments are required for this study protocol. An initial contact for enrollment will occur via a telephone call based on our recruitment and screening methods. Each site will be the primary contact for enrollment. Women meeting inclusion/exclusion criteria will sign a consent form. Consent forms will be mailed to potential participants with a pre-addressed envelope to send the signed consent back to the study coordinator. Participants will be given 24 or more hours to review the consent, prior to the consent interview. A copy of the signed consent will be retained for VA records. After consenting, women Veterans will be considered enrolled in the study. Enrolled participants will answer demographic questions and complete an ICIQ-UI baseline assessment. These questions will be collected on the phone by the site coordinator. The site coordinator will then contact the Birmingham site coordinator to provide demographic information and UI severity for randomization. The Birmingham site coordinator will enter the information into REDCap and provide the site coordinator with the randomization group. Once a participant is randomized, she will be contacted by the site coordinator and given a tutorial on MyHealtheBladder or VA Video Connect (VVC). Participants randomized to VVC will be scheduled for the 1:1 VVC visit with a Certified Registered Nurse Practitioners (CRNP's). Prior to starting the modality sessions, each randomization group will complete all the assessments through the secure internet portal (TONIC) and have the opportunity to complete over the phone (if needed). MyHealtheBladder will be composed of daily educational sessions related to bladder health, pelvic floor muscle exercises, and self-monitoring per usual care. The VVC visit will use printed materials in a booklet form (per usual care and in a recent clinical trial).[38] In Birmingham and Atlanta, a trained Certified Registered Nurse Practitioners (CRNPs) will deliver care via VVC using a standardized protocol. The Birmingham and Atlanta site CRNPs will perform the Durham VVC visits (204 total, including the non-responder visits) and provide this intervention remotely. After 8-weeks (Figure 2), the validated ICIQ-UI questionnaire will determine who is a responder or non-responder by meeting MCID criteria. Women who do not meet the MCID for the ICIQ-UI after 8-weeks will be considered "non-responders." The Birmingham VAMC team will randomize a second time for non-responders to continue the initial intervention or receive a remote VVC booster visit (Figure 2). Since the initial VVC visit and the additional VVC visit (adaptive randomization) will need to be scheduled, a double-blind study cannot be performed. Site investigative teams will be masked to all baseline, 8-week, 12-week, and 24-week/6-month outcomes.

Schedule of Primary, Secondary, and Key Variables Outcome Measures:

CONSTRUCTS	MEASURES AND KEY VARIABLES	Baseline	4-wks	8-wks	12-wks	6-mo
**PRIMARY OUTCOME**						
**Urinary Incontinence Severity/Symptom burden and Bother**	<u>International Consultation on Incontinence Modular Questionnaire – Urinary Incontinence Short Form</u>, **ICIQ-UI SF**, is a 3-item instrument that measures UI frequency, volume loss, and 1-item for bother[1] measured through the MyHealtheBladder intervention or VVC administration	X	X	X	X	X
**SECONDARY OUTCOMES**						

Other lower urinary tract symptoms: urgency, frequency, nocturia	International Consultation on Incontinence Modular Questionnaire (ICIQ)-Overactive Bladder (OAB) is a 4-item instrument that measures nocturia, urgency, frequency, urinary incontinence symptoms and bother[2]	X			X	X
Global Perceptions and Satisfaction with Treatment	Validated tools developed by our group for use in clinical trials of incontinence treatments[3]				X	X
Pelvic floor muscle exercise adherence	Self-reported adherence to behavioral therapy - collected weekly through the mHealth intervention[4]	X		X	X	X
Direct and indirect costs for UI	Incontinence Resource Utilization Questionnaire: IRUQ	X			X	
Miles saved	Map function with data to/from living location to the clinical sites (used as an outcome in our clinical demo CVT project)	X			X	
Usability	The Health Information Technology Usability Evaluation Scale (Health-ITUES) has demonstrated reliability and validity for use in assessing the usability of mHealth technologies in community-dwelling adults living with a chronic illness.[6]				X	
Adaptive Behavior Index	Validated tool – will only use the UI module[7]	X			X	
**COVARIATES**						
Demographics, including military sexual trauma	Age, race/ethnicity, marital status, living location, education, annual family income, military sexual trauma, and impairment from the PhenX Toolkit Measures, Tier 1	X				
Obstetric/Gynecologic (Health History)	Parity, type of delivery, hysterectomy, menopausal status	X				
Perceived Stress	PhenX Toolkit Measures, Psychosocial	X				
Other medical and mental health comorbidities	Comorbidity Index (CI; 36-item, include bipolar disorder).[8] Bipolar disorder diagnosis (based upon medical chart review). Blood tests (based upon medical chart review)--hemoglobin A1c or random glucose level, creatinine	X				X
Medications – focused only medications that may improve or worsen UI	Medical records and interview with participants: name, dose, frequency of use of prescribed and over-the-counter medications that affect UI, herbs, and supplements; medications known to affect bladder symptoms[9]	X			X	X
Overall sleep quality	Pittsburgh Sleep Quality Index (PSQI)[10] is one of the most widely-used self-report questionnaires for assessing sleep quality (during the past month).	X				
PUF	Pelvic Pain and Urgency/Frequency Patient Symptom Scale	X				
Treatment Follow-Up						X

"Annie" Satisfaction Questions	(For Annie Pilot Participants ONLY) 3-4-item questionnaire administered via "Annie" (i.e. text messaging) to gauge participant satisfaction with the text message reminders and motivational messages					X

Link for the PhenX Toolkit measures from above: https://www.phenxtoolkit.org/index.php

Interventions: Participants in both groups will receive our evidenced-based behavioral modality adapted into MyHealtheBladder or delivered through VVC visits.[7,43-45] The major components of the behavioral interventions include: bladder education on anatomy and function, pelvic floor muscle exercises with behavioral strategies, and self-monitoring. MyHealtheBladder also includes real-life stories and motivational quotes to reinforce behavior change, as well as reminder features for adherence. Figure 3 is a screen shot to portray the existing program on a mobile device. With this SMART design, we will compare the effectiveness of the 8-week MyHealtheBladder delivered daily to a 1:1 VVC visit that delivers similar behavioral content with oral instructions, a written booklet, and telephone follow-up visits. The adherence and reminder features will only be available to the women Veterans who are randomized to MyHealtheBladder. The reminders are sent daily to the participants. Built-in reminders are a core component of the evidenced-based intervention based on our pilot study data.

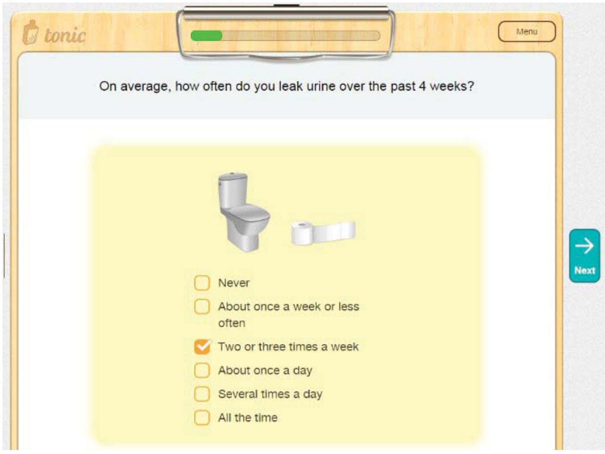

Participants expressed how helpful the daily reminders and the reminders when 3 sessions were missed, alerting them to go back and complete the sessions (Red Alert – reminders). Women will have the ability to complete the daily modules within 2 weeks from the missed sessions even if they did not complete them on the assigned day. The Birmingham site coordinators will monitor study sessions for all participants across the sites. Birmingham site coordinators will inform Durham and Atlanta site coordinators if a participant has not logged into MyHealtheBladder in the past 3 days or has not completed a data collection point within 3 days. Data collection for the primary outcome will occur through MyHealtheBladder and a similar online secure portal for women Veterans randomized to the VVC first. All entered data into MyHealtheBladder will be de-identified for review by the study coordinators. Women randomized to MyHealtheBladder and the VVC visit will have the primary outcome measured at baseline, 4-weeks, 8-weeks, and 24-weeks via the initial email link to the secure online portal through an internet survey or telephone call (based on preferences). They will also have a telephone call at 4-weeks, 8-weeks, 12-weeks, and 24-weeks to monitor adherence, help troubleshoot any problems, serve as a reminder to complete outcome measures. At 8-weeks, women who are non-responders to either the MyHealtheBladder or the VVC visit will be randomized to a 1:1 VVC visit or continue with self-management. Both randomization groups will have the primary outcome measured via telephone calls or internet survey at 12-weeks and at 24-weeks to assess durability.

Enrolled participants will have a chance to opt-in to receiving supplementary reminders and motivational messages via text message from a secure VA platform called "Annie". "Annie" was created by the VA Office of Connected Care. We aim to pilot test the use of "Annie" with up to 10 PRACTICAL participants at the Birmingham VA site.

Data Collection and Management: Baseline data will be collected via an internet survey on the web-based platform or via telephone (per Veteran preference) for all randomized women Veterans. Outcome data collection for the MyHealtheBladder will occur through the secure Tonic® platform at baseline, 4- and 8-weeks. Data collection for the VVC visit will occur through the same secure website (without the intervention contents) and/or via telephone at baseline, 4- and 8-weeks. Women will also answer questions pertaining to adherence to the exercises and strategies

through MyHealtheBladder and at 4, 8, 12, & 24 weeks for the VVC visit arms. At 12-weeks and 6-months, the final assessments will occur via an internet survey or via telephone (per Veteran preference) to capture any changes in medications, additional treatments, symptom burden, impact on quality of life, and other secondary measures. Data collected from the MyHealtheBladder, the internet surveys/questionnaires, and “Annie” satisfaction survey will be entered into a REDCap database created on the VA intranet by the Birmingham team. Any other completed data forms done via telephone will be scanned and placed into a secure drive specific for this study. The Birmingham team will then enter the information into REDCap. For security reasons, Birmingham (the coordinating center) will have access to the de-identified data for Atlanta and Durham. Atlanta and Durham will not have access to any de-identified data from the other participating sites.

Sample Size for Aims 1 and 2: Specifically, we powered this study and the sample size using the minimally important clinical difference (ICIQ-UI Short Form, Range is 0-21 and MICD is 2.52 (±2.56)) based on a Swedish study that had improvements in stress UI with an internet-based and mobile application-based pelvic floor muscle training intervention.[42] In this study, the authors reported a 3.9 point decrease (95% CI 3.0-4.7) in the ICIQ-UI SF score (11.1 ±2.8 to 7.0 ±3.5) with the mHealth intervention at 12-weeks. In our pilot data (n=21), we also used the ICIQ-UI SF and found a 4-point decrease from 12.6±3.9 at baseline to 8.7±4.0 at 8-weeks. Per one pragmatic clinical trial involving a nurse specialist for UI compared to usual care in a primary care setting (n=384), the authors found a decrease in UI severity from 11.1 ±4.3 to 9.1 (±2.9) using the ICIQ-UI SF at 12-weeks.[64] Compared to usual care, both groups meet the MICD for the ICIQ-UI SF.

Power and sample size calculations for SMART designs have more than one power function and follow a protocol detailed in Collins et al. (2014).[65] Initially, the sample size is based on the power to detect an effect size at the first stage of the trial. Given the UI severity reduction at 4-weeks cited above, the standardized effect size ranges from 0.40 to 0.45, resulting in total sample sizes ranging from 200 to 158 to have 80% power at a 5% significance level. The power to detect effects at the second stage of the SMART trial depends on the projected percentage of responders in each arm of the trial. Based on the UI severity reduction at 4-weeks, the percentage of responders could be as low as 40%; however, based on the UI severity reduction at 8-weeks the percentage of responders could be as high as 90%.

Based on a 40% response rate (achievement of the MICD at 8 weeks), 70% of patients in each arm would remain on their initially randomized treatment program and 30% in each arm would be re-randomized to the booster VVC visit or continued intervention. Based on an initial sample size of 200 patients completing the first stage, 140 (70 in each arm) would be available for comparison at the 12-week endpoint. This sample size would yield over 99% power to detect at a 5% significance level between arm differences based on the UI severity reduction at 8-weeks cited above. For a 1% significance level, the power would still be over 90%.

The power to detect the effect of the VVC booster within each arm is based on taking the 50% randomized to each arm and splitting them based on the percentage of responders. In this case of an initial sample size of 100 patients completing the first stage within each arm there would be 70 patients remaining on the initial treatment and 30 re-randomized to the VVC booster or continued intervention. For this sample size a standardized effect of 0.62 would need to be observed for 80% power at a 5% significance level. Based on a standard deviation of 4 this translates into the VVC booster changing UI severity by approximately 2.5 points, which is consistent with the MICD for the UI severity index. Based on the table (Table 3), a total sample size of 160 would provide adequate statistical power for detecting first-stage and second-stage between-arm effects. However, analyzing the within-arm effects of the VVC booster reduces the sample size by 50% and a **sample size of 200** is required.

Table 3. Power Estimates for First and Second Stage Power based on Standardized Effects Sizes (ES) and Projected Percentage of Responders.

Total Sample Size (N)	1st Stage Power m-Health vs VVC		Second Stage Power			Between-Arm Effect m-Health vs VVC		Within-Arm Effect of VVC Booster	
	ES=0.40	ES=0.45	Percent responders	Total N remaining	Total N re-randomized	ES=0.50	ES=0.60	ES=0.60	ES=0.65
N=200 (100 per arm)	80.4%	88.6%	40%	140	60	83.6%	94.1%	77.7%	83.9%
			50%	150	50	86.0%	95.5%	73.0%	79.6%
			60%	160	40	88.2%	96.5%	66.1%	73.1%
N=160 (80 per arm)	71.0%	80.8%	40%	112	48	74.6%	88.2%	68.0%	74.9%
			50%	120	40	77.5%	90.3%	63.1%	70.1%
			60%	128	32	80.1%	92.0%	56.4%	63.2%

Based on our pilot data and this pragmatic trial, we estimated that 200 women (100 in each arm) are needed for 80% power at 5% significance level to detect a difference of 2.52 (MICD) between the two groups. We anticipate a drop-out rate of 25% given our pilot data. To account for a 75% retention rate (25% drop-out rate), we will recruit up to 110 women at each site, **total sample size goal of 286 women**.

Data Analysis: The primary endpoint will be assessed at 8 and at 12 weeks with durability data collected at 24-weeks. Point estimates of mean ICIQ-UI SF change and standard deviation of change, as well as 95% CI will be performed using SAS statistical software (SAS Institute). Kolmogorov–Smirnov tests will be used to test normality of distributions. Comparability of groups at baseline will be analyzed using Chi-square test for categorical variables and two-sample t-test for continuous variables (if normally distributed) for demographics, medical history and number of medications. To test the overall differences in outcomes at 12-weeks, paired t-tests of differences will be used by summing results over both groups. Repeated measures ANOVA will be used to test outcome time differences from baseline between both groups, at 8-weeks, 12-weeks, and 24-weeks. Repeated measures ANCOVA will be used to adjust for baseline outcome measurements, UI type, age, BMI, parity, and depression.

Missing Data: Despite best efforts, there will inevitably be missing data to contend with in the analyses. Techniques we propose to use assume that missing data will be missing at random (MAR), meaning that the missingness mechanism does not depend on treatment success but may depend on baseline characteristics.[66] In general, we plan to include baseline factors that are associated with loss-to-follow-up in the analysis of outcomes. We also plan to evaluate the MAR assumption using sensitivity analysis.

Study Design for Aim 3 –Qualitative Analysis

Table 5. Key Domains for Interview Guides

Domain	Women Veterans	Providers
**Technology Access and Use**	**Usability; accessibility**	**Referring; Timing of care**
**Content**	**Education given daily vs booklet;**	**Expertise for UI care**
**Treatment Acceptance**	**Behavior change; relevance**	**Preferred delivery access method**
**Overall Evaluation**	**Expectations; impact**	**Ease of use in clinical setting**
**Dissemination Comments**	**Applicability; adding/removing**	**Unintended consequences**

Overview: Individual semi-structured interviews with women Veterans and providers at each clinical site will be conducted via telephone by investigators with expertise in Atlanta and Birmingham. The interviews will occur 7-10 days after the interventions are completed for the participants and at the end of the trial for the providers. Participants will include 3 separate groups based on the SMART design of this trial: women Veterans who complete the VVC only, women who have the MyHealtheBladder only, and women who have both interventions. We plan to conduct 72 interviews with women Veterans (48 at week 8 and an additional 24 at week 12) and 12 provider interviewers across the 3 sites. The research coordinator will sample potential participants by telephone (depending on recruitment strategy at each site) and coordinate the 45-minute to 1-hour interviews (based on our preliminary data) under the direction of the site PI and the qualitative team. Interviews will be conducted with providers who help enroll for the

study, i.e. women's health providers. We anticipate having 4 providers at each site, for 12 interviews (total). Expertise from The Salt Lake City Center of Innovation (COIN), led by Dr. Susan Zickmund, will provide support for transcription and coding.

Interviews: Each session will be conducted via telephone by a moderator trained in qualitative research principles (Dr. Katharina Echt and Dr. Anna Vandenberg, Birmingham/Atlanta GRECC). The interviewers will be the data collection instruments guided by well-designed semi-structured interview guides for the women Veterans and providers (see Appendices E and F). The interview guides have 5 sections and core questions with accompanying probes. The 5 domains mirror our pilot data (Table 5) from interviews, including technology access and use (barriers and supports), content provided (order, speed, preferences), treatment acceptance (understanding, use of pelvic floor muscle exercises, strategies), overall evaluation (expectations and impact), and suggestions for future dissemination (improve, remove, promotion).
Interviews will be audio-recorded. Field notes from the interviews will be maintained to contextualize the interview process. To manage the potential for data-based adjustments to the interview guides, biweekly updates will be reviewed and discussed within the qualitative implementation team conference calls. This will allow for modifications as indicated by the results of review of the field notes from the individual sessions. Examples may include discovering that specific technology is not understood by the participants or noting that a response may require more validation.

Data Collection and Management: The encrypted digital files will be uploaded by the site research coordinator to a secure centrally-located web-based shared site for transcription. The files will be transcribed verbatim using the Salt Lake City Centralized Transcription Service Program (CTSP). Transcription accuracy will be verified by a comparison of the audio files to the transcribed documents. Transcripts will be cleaned using a standardized protocol. The data analysis will only be conducted with the de-identified transcripts and will not refer back to the audio tapes. Transcription procedures and verification of the transcripts will be completed through the CTSP.

Data Analytic Approach: Initially we will perform a Directed Content Analysis of the transcripts. Directed Content Analysis begins with a conceptual framework for structuring the analysis, then utilize a deductive approach to explore textual data for insights relevant to the research question with the goal of validating and extending knowledge in the area of interest. Directed Content Analysis has particular utility in research areas where current theory needs further elucidation and description.[67] In this study, Directed Content Analysis will be used to identify 5 key domains specific to the structure of the interview guides and Table 5 above. These domains are related to barriers and incentives for change for participants and providers.
Our main subgroup analysis will be focused on type of intervention received with separate groups based on the two interventions. This means that for the purpose of coding, each interview will be identified by the women randomized to the MyHealtheBladder application compared to the VVC visit plus additional VVC visit per the SMART design. The general demographic descriptors for the make-up of the groups will also be included (e.g. race, ethnicity). This will allow us to consider the analysis process across interviews by age and general composition of the group. The transcribed texts of the interviews constitute the body of data that will undergo Directed Content Analysis. Specifically, all transcripts will be imported into Atlas.ti®, an on-line platform for qualitative data analysis designed to facilitate the organization and analysis of qualitative data.
Standard qualitative data analysis techniques will be used, beginning with coding and memoing.[68,69] Coding is a systematic process of disaggregating text and reclassifying it by categories that represent views and experiences repeated within and across interviews. Memoing entails making notations of researchers' conceptual and theoretical insights relating to the themes. Although it is part of the analytic process, memoing also plays an important role in the development and articulation of conceptual and theoretical frameworks during the interpretative phase of the study. As an adjunct, review of the field notes completed during the interview process will be completed to contextualize the interview data and identify any unique codes or concepts that may augment the initial coding scheme. Memoing by the analysis team will result in a preliminary codebook. The codebook will be comprised of the interview text and researcher memos that gave rise to the themes, including illustrative quotes exemplifying the theme. Each code will be designated

by name (typically using participant phrasing) and specified by an operational definition with inclusion and exclusion criteria. Variations within codes will generate subcodes. Patterns and associations across codes and coded text segments will be analyzed toward the development of thematic categories that indicate the relationship among codes.

The resulting thematic categories, will be organized into diagram structures or arrangements, that may be linear, sequential, circular, concentric or hierarchical (e.g., a tree diagram) in preparation for reporting.[70] Coders will be trained by Dr. Zickmund. Following the completion of this process, Dr. Zickmund will compile the resulting coding scheme and the definitions of the codes into a codebook. Staff will then use the codebook to code all transcripts. Coding disagreements will be resolved by research team consensus. The codebook may evolve and refinements may be made. Qualitative data analysis software Atlas.ti® will be used to organize transcripts, code, memo and analyze patterns and relationships across codes.[69] These data management and data analysis approaches meet the "Standards for Reporting Qualitative Research" as described by O'Brien et al for content analysis and grounded theory.[69,71,72]

IV. Schedule of Internet and Phone Visits

Telephone Visit	Timing	Schedule of Telephone visit goals, procedures, and assessments
	Pre-Enrollment	1. Site coordinator will complete Pre-Enrollment form with potential participant via telephone<br>2. Potential participant will review consent form via mail. Site coordinator will schedule a date/time to complete a consent interview.<br>3. Site coordinator will complete consent interview with potential participant on the phone, at scheduled time/date. Participant will sign form and mail back to VA, using a pre-addressed envelope.
1	Week 1	1. After consent form is received at VA site, site coordinator will have participant complete demographics and ICIQ-UI assessment on phone. Responses will be recorded via pen/paper by site coordinator. At the end of the phone call, site coordinator will setup a randomization phone call to occur within the next 7 business days.<br>2. Site coordinator will scan demographics and ICIQ-UI responses to Birmingham site coordinator within 24 hours of completion.
2	Reminder Call	1. Site coordinator will call participant 1 day prior to scheduled randomization visit to confirm participant will maintain the visit.
3	Week 2	1. Site coordinator will call Birmingham Site Coordinator for randomization assignment.<br>2. Once randomization assignment is received, site coordinator will contact participant and provide a tutorial for MyHealtheBladder or VVC visit<br>a. ALL participants will receive a tutorial on MyHealtheBladder/TONIC for data collection. Participants will be given 7 days to complete baseline assessments. If a participant has not started or completed baseline assessment prior to day 4, Birmingham site coordinator will inform the site coordinator and a reminder call will be placed. Following the VVC or MyHealtheBladder tutorial all participants should be reminded to complete the baseline assessments.<br>b. If participant randomized to VVC, site coordinator will provide tutorial to participant on VVC. Site coordinator will ensure participant has downloaded the software and has tested a mock call. Within 24 hours, site coordinator must contact the appropriate site to schedule the VVC visit. Durham site coordinator will contact Atlanta or Birmingham site coordinator to schedule VVC visit. Birmingham will contact Atlanta and Atlanta site coordinator will contact Birmingham site coordinator. The VVC must be scheduled within 10 days of the randomization visit. After the randomization visit with the participant is completed, site coordinator will mail the VVC booklet to the participant within 1-2 business days. Site coordinator will call participant after 3 days of mailing the VVC

		booklet to ensure receipt of the booklet, to confirm the future VVC appointment, and confirm completion of baseline measures.<br>c. If participant randomized to MyHealtheBladder, site coordinator will provide tutorial to participant. Site coordinator will ensure participant is able to log into MyHealtheBladder and navigate the modules. Site coordinator will provide instructions to participant about completing modules and answering questionnaires within the modules.<br>**NOTE**: Participants should not begin VVC or MyHealtheBladder program prior to completion of baseline assessments. Birmingham site coordinator will monitor MyHealtheBladder for completion rates.	
		**Study Arm: MyHealtheBladder**	**Study Arm: VVC**
4	Week 3	Goal: Veteran starts MyHealtheBladder content<br>Procedure:<br>1. Birmingham site coordinator will monitor MyHealtheBladder to ensure participant starts modules by day 3. If participant has not made progress through modules, Birmingham site coordinator will alert site coordinator.<br>2. Site coordinator will call participant to remind them of importance of completing modules. Site coordinator will also probe for any technical issues the participant may be having.<br>Assessments: None	Goal: Veteran completes VVC visit<br>Procedure:<br>1. Site coordinator places a reminder call to participant 1 day prior to VVC visit.<br>2. Participant will complete VVC visit with Birmingham or Atlanta continence provider.<br>Assessments: None
	Weeks 4-5	Goal: Monitor completion rates for daily MyHealtheBladder sessions<br>Procedure: Birmingham manages red alerts and will inform site RC for daily/weekly completion rates.<br>Assessments: None	Goal: Veteran continues self-management program.<br>Procedure: None<br>Assessments: None
5	Week 6	Goal: Monitor completion rates for daily MyHealtheBladder sessions<br>Procedure: Birmingham manages red alerts and will inform site RC for daily/weekly completion rates. Site coordinator completes telephone call at week 6 to remind participant to complete the 4-week ICIQ-UI assessment and adherence questions.<br>Assessments: ICIQ-UI; Adherence through MyHealtheBladder	Goal: Veteran continues self-management program.<br>Procedure: Site coordinator completes telephone call at week 6 to remind participant to complete the 4-week ICIQ-UI assessment and adherence questions.<br>Assessments: ICIQ-UI; Adherence
	Weeks 7-9	Goal: Monitor completion rates for daily MyHealtheBladder sessions<br>Procedure: Birmingham manages red alerts and will inform site RC for daily/weekly completion rates.<br>Assessments: None	Goal: Veteran continues self-management program.<br>Procedure: None<br>Assessments: None
6	Week 10	Goal: Monitor daily/weekly MyHealtheBladder sessions; MyHealtheBladder daily program finished.<br>Procedure: Birmingham manages red alerts, informs site RC for daily/weekly completion rates, and alerts site RC to adaptive randomization assessment.	Goal: Veteran continues self-management program.<br>Procedure<br>1. Site coordinator will place reminder call to participant for completion of Week 8 ICIQ-UI assessment. Participant will complete ICIQ-UI assessment.

		1. Site coordinator will place reminder call to participant for completion of Week 8 ICIQ-UI assessment. Participant will complete ICIQ-UI assessment.<br>2. Birmingham site coordinator notify site coordinator if participant is a responder or non-responder based on ICIQ-UI results.<br>3. If non-responder, participant receives adaptive re-randomization assignment from Birmingham site coordinator. Site coordinator will call participant and inform them of the assignment. If randomized to VVC, site coordinator will follow guidelines from "Week 2 Section 2b" to setup VVC visit. If randomized to self-management, site coordinator will contact participant to inform them to continue skills learned during the 1st 8 weeks.<br>4. If responder, participant will be a completer. Site coordinator will ask participant if they would like to participate in qualitative interview. If participant agrees, participant will complete a qualitative interview within 7 days; scheduled by site coordinator. Site coordinator will notify Birmingham coordinator for tracking purposes. If a responder and selected, participant will complete qualitative interview with team interviewer. Site RC will contact qualitative interviews, to determine open time slot for 30-minute interview. When day/time agreed upon by participant and interviewer, site RC will inform Birmingham RC for tracking purposes.<br>Assessments: ICIQ-UI; Adherence through MyHealtheBladder	2. Birmingham site coordinator notify site coordinator if participant is a responder or non-responder based on ICIQ-UI results.<br>3. If non-responder, participant receives adaptive re-randomization assignment from Birmingham site coordinator. Site coordinator will call participant and inform them of the assignment. If randomized to VVC, site coordinator will follow guidelines from "Week 2 Section 2b" to setup VVC visit. If randomized to self-management, site coordinator will contact participant to inform them to continue skills learned during the 1st 8 weeks.<br>4. If responder, participant will be a completer. Site coordinator will ask participant if they would like to participate in qualitative interview. If participant agrees, participant will complete a qualitative interview within 7 days; scheduled by site coordinator. Site coordinator will notify Birmingham coordinator for tracking purposes.<br>Assessments: ICIQ-UI; Adherence Assessments: ICIQ-UI; adherence
**Adaptive Re-Randomization**			
7	Week 11	Goal: Adaptive Re-Randomization – Veteran does self-management and has a VVC visit, if symptoms not improved (non-responders)<br>Procedure:<br>1. Non-responder participant will begin adaptive re-randomization.<br>Assessments: None	Goal: Adaptive Re-Randomization – Veteran does VVC visit, if symptoms not improved (non-responders)<br>Procedure:<br>1. Site coordinator complete reminder call to participant 1 day prior to scheduled visit.<br>2. Non-responder participant completes VVC visit with continence provider within 10 days of adaptive re-randomization.<br>Assessments: None
**1st follow-up visit (Week 14)**			
8	Week 12-14	Goal: Continue self-management<br>Procedure:<br>1. Site coordinator will complete reminder call to participant for 12-Week assessments. Participants will also receive an email reminder from MyHealtheBladder to complete assessments.<br>2. Birmingham site coordinator will alert site coordinator if participant has not completed	Goal: Veteran continues self-management.<br>Procedure:<br>1. Site coordinator will complete reminder call to participant for 12-Week assessments. Participants will also receive an email reminder from TONIC to complete assessments.<br>2. Birmingham site coordinator will alert site coordinator if participant has not completed

		12-week assessments within 3 days. Site coordinator will place a 2nd reminder call to participant. Participant will be given 7 days to complete assessments.<br>3. If <u>non-responder</u>, participant will be a completer. Site coordinator will ask participant if they would like to participate in qualitative interview. If participant agrees, participant will complete a qualitative interview within 7 days; scheduled by site coordinator. Site coordinator will notify Birmingham coordinator for tracking purposes.<br><u>Assessments:</u> 12-week assessments; Adherence through MyHealtheBladder	12-week assessments within 3 days. Site coordinator will place a 2nd reminder call to participant. Participant will be given 7 days to complete assessments.<br>3. If <u>non-responder</u>, participant will be a completer. Site coordinator will ask participant if they would like to participate in qualitative interview. If participant agrees, participant will complete a qualitative interview within 7 days; scheduled by site coordinator. Site coordinator will notify Birmingham coordinator for tracking purposes.<br><u>Assessments:</u> 12-week assessments; Adherence
	Weeks 15-23	Goal: Veteran continues self-management<br><u>Procedure</u>: None<br><u>Assessments:</u> None	
**Final Follow-up Visit (24 weeks)**			
9	Week 24	Goal: Finalize study assessments<br><u>Procedure:</u><br>1. Site coordinator will complete reminder call to participant for 24-Week assessments. Participants will also receive an email reminder from MyHealtheBladder to complete assessments.<br>2. Birmingham site coordinator will alert site coordinator if participant has not completed 24-week assessments within 3 days. Site coordinator will place a 2nd reminder call to participant. Participants will have 14 days to complete 24-week assessments.<br><u>Assessments</u>: 24-week assessments; Adherence	

Protocol Abbreviations

3-P	Predisposing, Precipitating, and Perpetuating
ANCOVA	Analysis of Covariance
ANOVA	Analysis of Variance
ATL	Atlanta
BIR	Birmingham
BMI	Body Mass Index
CBOC	Community Based Outpatient Clinic
CDW	Corporate Data Warehouse
COIN	Center of Innovation
CRNP	Certified Registered Nurse Practitioner
CTSP	Centralized Transcription Service Program
CVT	Clinical Video Telehealth
DUR	Durham
GRECC	Geriatrics Research, Education, and Clinical Center
Health-ITUES	Health Information Technology Usability Evaluation Scale
HSR&D	Health Services Research and Development
ICIQ-OAB	International Consultation on Incontinence Modular Questionnaire – Overactive Bladder
ICIQ-UI SF	International Consultation on Incontinence Modular Questionnaire - Urinary Incontinence Short Form
MAR	Missing at Random
m-health	Mobile Health
MICD	Minimal Clinically Important Difference
ORD	Office of Research and Development
PFMT	Pelvic Floor Muscle Training
PSQI	Pittsburgh Sleep Quality Index
PTSD	Post-traumatic Stress Disorder
REDCap	Research Electronic Data Capture
SMART	Sequential, multiple assignment, randomized trial
TBI	Traumatic Brain Injury
UI	Urinary Incontinence
VAMC	VA Medical Center
VHA	VA Health Administration
VVC	VA Video Connect
WH-PBRN	Women's Health Practice Based Research Network
