# Supplementary material for: Remote Access to Urinary Incontinence Treatments for Women Veterans: The PRACTICAL Randomized Clinical Trial
Source: JAMA Netw Open. 2025 Sep 16;8(9):e2532111. doi: 10.1001/jamanetworkopen.2025.32111 (PMC12441875; doi:10.1001/jamanetworkopen.2025.32111)
Supplement: Supplement 2. — eTable 1. Baseline Descriptive Data for Responders and Non-Responders at 8-Weeks eTable 2. Sensitivity Analyses: Multiple Imputation and Linear Mixed Model Analyses of Between Group Changes in ICIQ-UI Scores eFigure. ICIQ-UI SF Score Change by Visit [file jamanetwopen-e2532111-s002.pdf]

Supplementary Online Content

Markland AD, Goldstein KM, Beasley TM, et al. Optimizing remote access to urinary incontinence treatments for women veterans (PRACTICAL): a randomized clinical trial. *JAMA Netw Open*. 2025;8(9):e2532111. doi:10.1001/jamanetworkopen.2025.32111

eTable 1. Baseline Descriptive Data for Responders and Non-Responders at 8-Weeks

eTable 2. Sensitivity Analyses: Multiple Imputation and Linear Mixed Model Analyses of Between Group Changes in ICIQ-UI Scores

eFigure. ICIQ-UI SF Score Change by Visit

This supplementary material has been provided by the authors to give readers additional information about their work.

© 2025 Markland AD et al. *JAMA Network Open*.

eTable 1. Baseline Descriptive Data for Responders and Non-Responders at 8-Weeks

	MyHealtheBladder (MHB) N=84						Video Visit via VA Video Connect (VVC) N=104					
				Non-Responders Re-Randomization n=22/29						Non-Responders Re-Randomization n=58/61		
	MHB Responders, No (%)	MHB Non-Responders, No (%)	p	MHB Continue, No (%)	MHB & VVC Booster, No (%)	p	VVC Responders. No (%)	VVC Non-Responders, No (%)	p	VVC Continue, No (%)	VVC & VVC Booster, No (%)	p
**N**	**55**	**29**	**value**	**10**	**12**		**43**	**61**		**31**	**27**	
**Age (years)** Mean (95% CI)	56.6 (54.0-59.2)	52.6 (48.4-56.7)	0.10	57.1 (49.3- 64.9)	50.4 (55.9-49.5)	0.13	54.3 (50.4-58.3)	52.7 (50.0-55.5)	0.51	53.2 (49.3-57.1)	51.0 (46.6-55.4)	0.45
**Age, range**	35 - 75	27 - 74	0.11	38 - 74	34 - 64	0.12	23 - 83	24 - 77	0.43	24 - 77	31 - 71	0.51
**Ethnicity**			0.09			0.10			0.21			0.64
Hispanic	2 (3.6%)	0 (0.0%)		0 (0.0%)	0 (0%)		5 (11.6%)	3 (4.9%)		2 (6.5%)	1 (3.7%)	
Not Hispanic	53 (96.4%)	27 (93.1%)		8 (80.0%)	12 (100%)		37 (86.0%)	58 (95.1%)		29 (93.5%)	26 (96.3%)	
Not Reported	0 (0.0%)	2 (6.9%)		2 (20.0%)	0 (0.0%)		1 (2.3%)	0 (0.0%)		0 (0.0%)	0 (0.0%)	
**Race**			0.79			0.64			0.84			0.47
White	17 (30.9%)	7 (24.1%)		3 (30.0%)	3 (25.0%)		17 (39.5%)	26 (42.6%)		12 (38.7%)	14 (51.9%)	
Black/African American	35 (63.6%)	21 (72.4%)		7 (70.0%)	8 (66.7%)		21 (48.8%)	31 (50.8%)		17 (54.8%)	11 (40.7%)	
More than one	2 (3.6%)	1 (3.4%)		0 (0.0%)	1 (8.3%)		1 (2.3%)	1 (1.6%)		0 (0.0%)	1 (3.7%)	
Other	1 (1.8%)	0 (0.0%)		0 (0.0%)	0 (0.0%)		4 (9.3%)	3 (4.9%)		2 (6.5%)	1 (3.7%)	
**Education**			0.07			0.44			0.59			0.85
HS graduate	1 (1.8%)	2 (6.9%)		1 (10.0%)	0 (0.0%)		1 (2.3%)	4 (6.6%)		3 (9.7%)	1 (3.7%)	

© 2025 Markland AD et al. *JAMA Network Open*.

Some college	4 (7.3%)	7 (24.1%)		1 (10.0%)	4 (33.3%)		8 (18.6%)	13 (21.3%)		6 (19.4%)	6 (22.2%)	
Associate degree	19 (34.5%)	4 (13.8%)		1 (10.0%)	0 (0.0%)		9 (20.9%)	10 (16.4%)		5 (16.1%)	3 (11.1%)	
Bachelor's degree	20 (36.4%)	9 (31.0%)		4 (40.0%)	5 (41.7%)		13 (30.2%)	23 (37.7%)		12 (38.7%)	11 (40.7%)	
Graduate degree	11 (20.0%)	7 (24.1%)		3 (30.0%)	3 (25.0%)		12 (27.9%)	11 (18.0%)		5 (16.1%)	6 (22.2%)	
**Marital Status**			0.98			0.06			0.35			0.14
Married	23 (41.8%)	13 (44.8%)		4 (40.0%)	5 (41.7%)		14 (32.6%)	22 (36.1%)		13 (41.9%)	9 (33.3%)	
Divorced	16 (29.1%)	8 (27.6%)		5 (50.0%)	2 (16.7%)		18 (41.9%)	21 (34.4%)		6 (19.4%)	13 (48.1%)	
Widowed	3 (5.5%)	1 (3.4%)		0 (0.0%)	0 (0.0%)		2 (4.7%)	4 (6.6%)		3 (9.7%)	1 (3.7%)	
Separated	3 (5.5%)	1 (3.4%)		1 (10.0%)	0 (0.0%)		0 (0.0%)	3 (4.9%)		2 (6.5%)	0 (0.0%)	
Never married	10 (18.2%)	6 (20.7%)		0 (0.0%)	5 (41.7%)		7 (16.3%)	11 (18.0%)		7 (22.6%)	4 (14.8%)	
Unmarried couple	0 (0.0%)	0 (0.0%)		0 (0.0%)	0 (0.0%)		2 (4.7%)	0 (0.0%)		0 (0.0%)	0 (0.0%)	
**Branch of Service**			0.35			0.14			0.88			0.17
Army	35 (63.6%)	15 (51.7%)		4 (40.0%)	8 (66.7%)		21 (48.8%)	34 (55.7%)		15 (48.4%)	17 (63.0%)	
National Guard	0 (0.0%)	1 (3.4%)		0 (0.0%)	0 (0.0%)		2 (4.7%)	2 (3.3%)		1 (3.2%)	1 (3.7%)	
Navy	5 (9.1%)	5 (17.2%)		1 (10.0%)	3 (25.0%)		7 (16.3%)	7 (11.5%)		6 (19.4%)	1 (3.7%)	
Air Force	8 (14.5%)	3 (10.3%)		3 (30.0%)	0 (0.0%)		10 (23.3%)	11 (18.0%)		7 (22.6%)	4 (14.8%)	
NOAA	0 (0.0%)	0 (0.0%)		0 (0.0%)	0 (0.0%)		0 (0.0%)	1 (1.6%)		0 (0.0%)	1 (3.7%)	
Marine Corps	0 (0.0%)	0 (0.0%)		0 (0.0%)	0 (0.0%)		1 (2.3%)	3 (4.9%)		0 (0.0%)	3 (11.1%)	
Coast Guard	0 (0.0%)	1 (3.4%)		0 (0.0%)	0 (0.0%)		0 (0.0%)	1 (1.6%)		1 (3.2%)	0 (0.0%)	
Reserve	7 (12.7%)	4 (13.8%)		2 (20.0%)	1 (8.3%)		2 (4.7%)	2 (3.3%)		1 (3.2%)	0 (0.0%)	
**Years of Service** Mean (95% CI)	10.6 (8.3-13.0)	7.6 (5.1-10.2)	0.08	8.5 (2.7-14.3)	7.5 ± 6.0	0.75	8.9 (6.3-11.5)	9.7 (7.7-11.7)	0.63	10.2 (7.1-13.3)	9.9 (6.9-12.8)	0.88

© 2025 Markland AD et al. *JAMA Network Open*.

Median (IQR)	6.25 (4,20)	5.5 (3,9)	0.14	5.75 (4,8)	5 (3.5,12)	0.84	4 (3,12)	7 (4,15)	0.45	7 (4,16)	9 (4,15)	0.96
**BMI (kg/m²)**<br>Mean ± SD	32.1<br>(30.4-33.8)	32.5<br>(29.6-35.4)	0.80	34.2<br>(26.9-41.5)	32.5<br>(28.4-36.6)	0.65	32.8 ± 6.7	31.5<br>(29.7-33.2)	0.33	31.1<br>(28.8-33.4)	31.0<br>(28.6-33.5)	0.97
Median (IQR)	31 (27,36)	31 (27,36)	0.95	30 (26,43)	31 (30,35)	0.90	32 (27,37)	32 (26,37)	0.41	33 (25,37)	32 (28,36)	0.89
**Self-reported Characteristics**												
**Comorbidities**<br>Mean ± SD	8.5<br>(7.7-9.3)	8.5<br>(7.3-9.7)	0.99	6.8<br>(4.7-8.9)	8.8<br>(7.1-10.4)	0.12	7.6<br>(6.6-8.5)	7.3<br>(6.5-8.2)	0.73	7.5<br>(28.6-33.5)	7.0<br>(5.5-8.5)	0.61
Median (IQR)	8 (6,11)	8 (6,10)	0.84	6 (5,8)	8 (7,10)	0.07	7 (5,10)	7 (5,10)	0.87	7 (6,10)	7 (3,10)	0.60
**Diabetes N (%)**	7 (12.7%)	4 (13.8%)	0.89	1 (10.0%)	3 (25.0%)	0.36	4 (9.3%)	8 (13.1%)	0.55	4 (12.9%)	3 (11.1%)	0.83
**Medications for UI**	7 (12.7%)	1 (3.4%)	0.17	1 (10.0%)	0 (0.0%)	0.26	3 (7.0%)	4 (6.6%)	0.93	2 (6.5%)	2 (7.4%)	0.89
**Depression N (%)**	42 (76.4%)	21 (72.4%)	0.69	6 (60.0%)	9 (75.0%)	0.45	30 (69.8%)	44 (72.1%)	0.79	23 (74.2%)	18 (66.7%)	0.53
**PTSD**	28 (50.9%)	18 (62.1%)	0.33	5 (50.0%)	8 (66.7%)	0.43	18 (41.9%)	31 (50.8%)	0.37	13 (41.9%)	16 (59.3%)	0.19
**Military Sexual Trauma**			0.42			0.14			0.54			0.80
No	34 (61.8%)	15 (51.7%)		7 (70.0%)	4 (33.3%)		23 (53.5%)	28 (45.9%)		16 (51.6%)	12 (44.4%)	
Yes	16 (29.1%)	11 (37.9%)		2 (20.0%)	7 (58.3%)		16 (37.2%)	23 (37.7%)		11 (35.5%)	10 (37.0%)	
Don't Know	0 (0.0%)	1 (3.4%)		1 (10.0%)	0 (0.0%)		0 (0.0%)	0 (0.0%)		0 (0.0%)	0 (0.0%)	
Did Not Respond	5 (9.1%)	2 (6.9%)		0 (0.0%)	1 (8.3%)		4 (9.3%)	10 (16.4%)		4 (12.9%)	5 (18.5%)	
**Hysterectomy**	29 (52.7%)	10 (34.5%)	0.11	4 (40.0%)	4 (33.3%)	0.75	17 (39.5%)	26 (42.6%)	0.75	11 (35.5%)	13 (48.1%)	0.33
**Post-menopause**	45 (81.8%)	16 (55.2%)	0.009	8 (80.0%)	5 (41.7%)	0.07	28 (65.1%)	38 (62.3%)	0.77	19 (61.3%)	17 (63.0%)	0.90
**Parity**			0.77			0.56			0.60			0.47
None	5 (9.1%)	1 (3.4%)		1 (10.0%)	0 (0.0%)		9 (20.9%)	9 (14.8%)		6 (19.4%)	3 (11.1%)	

© 2025 Markland AD et al. *JAMA Network Open*.

One	11 (20.0%)	4 (13.8%)		1 (10.0%)	1 (8.3%)		9 (20.9%)	11 (18.0%)		5 (16.1%)	5 (18.5%)	
Two	15 (27.3%)	10 (34.5%)		2 (20.0%)	6 (50.0%)		8 (18.6%)	17 (27.9%)		10 (32.3%)	7 (25.9%)	
Three	14 (25.5%)	9 (31.0%)		4 (40.0%)	3 (25.0%)		10 (23.3%)	10 (16.4%)		2 (6.5%)	6 (22.2%)	
Four or More	10 (18.2%)	5 (17.2%)		2 (20.0%)	2 (16.7%)		7 (16.3%)	14 (23.0%)		8 (25.8%)	6 (22.2%)	
**Device Types – Not Mutually Exclusive**												
Cell phone	43 (78.2%)	22 (75.9%)	0.81	7 (70.0%)	9 (75.0%)	0.79	35 (81.4%)	50 (82.0%)	0.94	22 (71.0%)	25 (92.6%)	0.04
Tablet	20 (36.4%)	13 (44.8%)	0.45	4 (40.0%)	5 (41.7%)	0.94	16 (37.2%)	20 (32.8%)	0.64	9 (29.0%)	9 (33.3%)	0.72
Laptop/PC	39 (70.9%)	14 (48.3%)	0.04	6 (60.0%)	5 (41.7%)	0.39	29 (67.4%)	46 (75.4%)	0.37	23 (74.2%)	21 (77.8%)	0.75
**UI Severity**<br>**Mean ± SD**	12.5<br>(11.3-13.7)	10.0<br>(8.3-11.8)	0.02	10.0<br>(6.8-13.2)	9.3<br>(6.1-12.4)	0.71	12.1<br>(11.1-13.2)	10.2<br>(9.2-11.2)	0.009	10.6<br>(9.2-12.0)	9.6<br>(8.0-11.1)	0.32
**Severity of UI, categories**			0.001			0.95			0.03			0.34
Mild (1-5)	0	5 (17.2%)	0.001*	0	0	0.76*	0	7 (11.5%)	0.01*	2 (6.5%)	1 (3.7%)	0.19*
Moderate (6-12)	28 (50.9%)	18 (62.1%)		8 (80.0%)	12 (100%)		21 (48.8%)	34 (55.7%)		29 (93.5%)	26 (96.3%)	
Severe (13-21)	27 (49.1%)	6 (20.7%)		2 (20.0%)	0		22 (51.2%)	20 (32.8%)		0	0	
**UI Type – Not Mutually Exclusive**												
Leaks before toileting	47 (85.5%)	24 (82.8%)	0.75	8 (80.0%)	10 (83.3%)	0.84	37 (86.0%)	53 (86.9%)	0.90	25 (80.6%)	25 (92.6%)	0.19
Leaks with cough/sneeze	44 (80.0%)	20 (69.0%)	0.26	9 (90.0%)	7 (58.3%)	0.10	31 (72.1%)	52 (85.2%)	0.10	27 (87.1%)	23 (85.2%)	0.83
Leaks when asleep	24 (43.6%)	12 (41.4%)	0.84	6 (60.0%)	5 (41.7%)	0.39	9 (20.9%)	20 (32.8%)	0.18	12 (38.7%)	6 (22.2%)	0.18

© 2025 Markland AD et al. *JAMA Network Open*.

Leaks with exercise/activity	37 (67.3%)	21 (72.4%)	0.63	8 (80.0%)	8 (66.7%)	0.48	28 (65.1%)	45 (73.8%)	0.34	21 (67.7%)	22 (81.5%)	0.23
Leaks after urination	27 (49.1%)	17 (58.6%)	0.41	8 (80.0%)	6 (50.0%)	0.15	13 (30.2%)	32 (52.5%)	0.02	18 (58.1%)	12 (44.4%)	0.30
Leaks for no obvious reason	28 (50.9%)	13 (44.8%)	0.60	6 (60.0%)	5 (41.7%)	0.39	16 (37.2%)	31 (50.8%)	0.17	16 (51.6%)	14 (51.9%)	0.99
Leaks all the time	12 (21.8%)	6 (20.7%)	0.90	4 (40.0%)	1 (8.3%)	0.08	2 (4.7%)	12 (19.7%)	0.03	8 (25.8%)	3 (11.1%)	0.15

* Cochran-Armitage Trend Test

© 2025 Markland AD et al. *JAMA Network Open*.

eTable 2. Sensitivity Analyses: Multiple Imputation and Linear Mixed Model Analyses of Between Group Changes in ICIQ-UI Scores

	**Multiple Imputation Analysis***				**Linear Mixed Model Analysis#**			
	**My Healthy Bladder (MHB)**	**VA Video Connect (VVC)**	**Between-Arm Difference**		**My Healthy Bladder (MHB)**	**VA Video Connect (VVC)**	**Between-Arm Difference**	
**Study Visit**	**Mean (95% CI)**	**Mean (95% CI)**	**(95% CI)**	**p-value**	**Mean (95% CI)**	**Mean (95% CI)**	**(95% CI)**	**p-value**
Baseline	11.6 (9.9 – 13.3)	11.0 (10.3 – 11.7)	0.6 (-0.4 – 1.6)	0.2457	11.2 (9.5 – 12.9)	10.7 (9.1 – 12.4)	0.5 (-0.5 – 1.4)	0.3460
4-weeks	8.7 (7.7 – 9.7)	10.2 (9.3 – 11.0)	-1.5 (-2.7 – -0.2)	0.0215	8.4 (6.6 – 10.3)	9.5 (7.8 – 11.1)	-1.0 (-2.2 – 0.1)	0.0864
**4-week change from baseline**	-2.9 (-3.8 – -2.0)	-0.8 (-1.6 – -0.1)	-2.1 (-3.1 – -1.0)	0.0002	-2.7 (-3.5 – -2.0)	-1.3 (-1.9 – -0.6)	-1.5 (-2.5 – -0.5)	0.0042
8-weeks	8.1 (7.1 – 9.1)	9.3 (8.5 – 10.1)	-1.2 (-2.4 – 0.0)	0.0567	7.8 (6.0 – 9.6)	8.8 (7.1 – 10.5)	-1.0 (-2.2 – 0.2)	0.1033
**8-week change from baseline**	-3.5 (-4.4 – -2.6)	-1.7 (-2.5 – -1.0)	-1.8 (-3.0 – -0.6)	0.0033	-3.4 (-4.3 – -2.5)	-1.9 (-2.6 – -1.3)	-1.5 (-2.5 – -0.4)	0.0094
12-weeks	8.1 (7.3 – 9.0)	9.1 (8.2 – 9.9)	-0.9 (-2.1 – 0.2)	0.1188	7.7 (5.9 – 9.5)	8.5 (6.8 – 10.2)	-0.8 (-2.0 – 0.4)	0.2145
**12-week change from baseline (Primary outcome)**	-3.5 (-4.2 – -2.7)	-1.9 (-2.7 – -1.2)	-1.5 (-2.6 – -0.5)	0.0042	-3.5 (-4.2 – -2.7)	-2.3 (-3.0 – -1.5)	-1.2 (-2.3 – -0.2)	0.0233
24-weeks	7.9 (6.8 – 9.1)	8.3 (7.4 – 9.3)	-0.4 (-1.9 – 1.1)	0.5952	7.4 (5.6 – 9.3)	7.6 (5.9 – 9.3)	-0.2 (-1.4 – 1.1)	0.8068
24-week change from baseline (secondary outcome)	-3.7 (-4.8 – -2.6)	-2.7 (-3.6 – -1.8)	-1.0 (-2.4 – 0.4)	0.1685	-3.8 (-4.7 – -2.9)	-3.1 (-3.9 – -2.3)	-0.6 (-1.8 – 0.6)	0.2988

* Group Means with 95% CIs and Between-Arm Differences with 95% CIs based on ANOVA performed on scores at each week or on change scores at each week. Estimates based on 10 imputed data sets.

# Estimates from Linear Mixed Model using heteroscedastic unstructured covariances with race as a covariate and site as a random effect.

NOTE: Minimal Important Clinical Difference (MICD) is **2.52** for the ICIQ-UI. Study designed for primary outcome is at 12-weeks with a decrease of the ICIQ-UI by 2.52 points.

© 2025 Markland AD et al. *JAMA Network Open*.

eFigure. ICIQ-UI SF Score Change by Visit

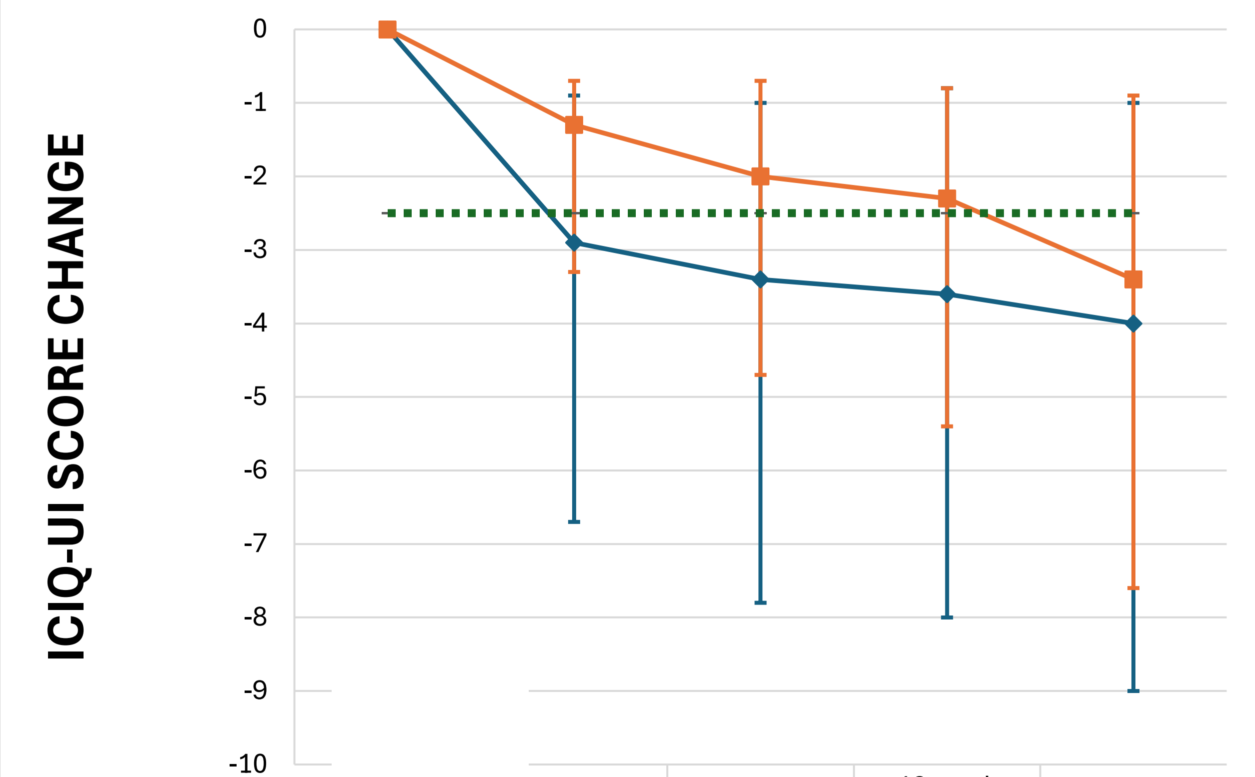

	Baseline	4-week change from baseline	8-week change from baseline	12-week change from baseline (Primary outcome)	24-week change from baseline
N (MHB)	137	85	84	100	92
N (VVC)	149	102	104	100	86
MHB	0	-2.9	-3.4	-3.6	-4
VVC	0	-1.3	-2	-2.3	-3.4
ICIQ-UI MCID	-2.5	-2.5	-2.5	-2.5	-2.5

© 2025 Markland AD et al. *JAMA Network Open*.
