# Supplementary material for: Remote Access to Urinary Incontinence Treatments for Women Veterans: The PRACTICAL Randomized Clinical Trial
Source: JAMA Netw Open. 2025 Sep 16;8(9):e2532111. doi: 10.1001/jamanetworkopen.2025.32111 (PMC12441875; doi:10.1001/jamanetworkopen.2025.32111)
Supplement: Supplement 3. — Data Sharing Statement [file jamanetwopen-e2532111-s003.pdf]

Data Sharing Statement

Markland. Optimizing Remote Access to Urinary Incontinence Treatments for Women Veterans (PRACTICAL). *JAMA Netw Open*. Published September 16, 2025.
doi:10.1001/jamanetworkopen.2025.32111

Data
Data available: Yes
Data types: Deidentified participant data, Data dictionary
How to access data: alayne.markland@va.gov
When available: With publication

Supporting Documents
Document types: Other (please specify)
Additional Information: Protocol
How to access documents: alayne.markland@va.gov
When available: With publication

Additional Information
Who can access the data: Researchers with approval
Types of analyses: for any purpose
Mechanisms of data availability: Approval of proposal and signed data access agreement
Any additional restrictions: NA
